# Su(Hw) interacts with Combgap to establish long-range chromatin contacts

**DOI:** 10.1186/s13072-024-00541-x

**Published:** 2024-05-21

**Authors:** Nadezhda E. Vorobyeva, Alexey N. Krasnov, Maksim Erokhin, Darya Chetverina, Marina Mazina

**Affiliations:** 1 Institute of Gene Biology, Russian Academy of Sciences, Moscow, 119334 Russia; 2 Center for Precision Genome Editing and Genetic Technologies for Biomedicine, Institute of Gene Biology, Russian Academy of Sciences, Moscow, 119334 Russia

**Keywords:** Insulator binding protein, Su(Hw), Combgap, Chromatin architecture, Transcription, Long-range interactions

## Abstract

**Background:**

Insulator-binding proteins (IBPs) play a critical role in genome architecture by forming and maintaining contact domains. While the involvement of several IBPs in organising chromatin architecture in *Drosophila* has been described, the specific contribution of the Suppressor of Hairy wings (Su(Hw)) insulator-binding protein to genome topology remains unclear.

**Results:**

In this study, we provide evidence for the existence of long-range interactions between chromatin bound Su(Hw) and Combgap, which was first characterised as Polycomb response elements binding protein. Loss of Su(Hw) binding to chromatin results in the disappearance of Su(Hw)-Combgap long-range interactions and in a decrease in spatial self-interactions among a subset of Su(Hw)-bound genome sites. Our findings suggest that Su(Hw)-Combgap long-range interactions are associated with active chromatin rather than Polycomb-directed repression. Furthermore, we observe that the majority of transcription start sites that are down-regulated upon loss of Su(Hw) binding to chromatin are located within 2 kb of Combgap peaks and exhibit Su(Hw)-dependent changes in Combgap and transcriptional regulators’ binding.

**Conclusions:**

This study demonstrates that Su(Hw) insulator binding protein can form long-range interactions with Combgap, Polycomb response elements binding protein, and that these interactions are associated with active chromatin factors rather than with Polycomb dependent repression.

**Supplementary Information:**

The online version contains supplementary material available at 10.1186/s13072-024-00541-x.

## Background

Understanding how chromatin architecture contributes to the correct spatio-temporal program of cell development is an important issue under active investigation in chromatin biology. Recent advances in chromosome conformation capture techniques have provided valuable insights into the organisation of genome architecture. However, it remains unclear how chromatin architecture is established and translated into a cell-specific developmental program.

Insulator-binding proteins (IBPs) play pivotal role among the various forces and mechanisms that contribute to shaping and maintaining of genome topology [1, 2]. In *Drosophila*, several IBPs enriched in topologically associated domain (TAD) borders have been identified [3–7]. Recent studies suggest that distinct IBP motifs and their combinations can define TAD borders [8]. Since many *Drosophila* IBPs exhibit properties of homo- or hetero-oligomerisation [3, 9], it has been suggested that these IBPs could maintain TAD borders through the formation of long-range interactions (LRIs) between them. However, the importance of IBPs for establishing TAD borders might be overestimated. Recent studies demonstrated that dCTCF and BEAF32 knockdowns disrupt only a relatively small number of TAD borders [7, 8]. The contribution of these proteins to gene expression regulation may not only be due to the maintenance of TAD borders but also to the formation of specific regulatory long-range chromatin interactions between non-border IBP sites [7, 10].

IBPs can influence transcription by determining the nuclear or genomic context to which IBP-bound chromatin is exposed. For example, dCTCF promotes both the localisation of repressed transgene to Polycomb bodies and the recruitments of active transgenes to transcription factories [11]. BEAF32 can regulate the genomic context of chromatin regions with which it interacts, facilitating the formation of H3K27me3 micro-domains in euchromatin regions [10]. IBPs frequently localise next to Polycomb-response elements (PREs) [12, 13]. Recent studies suggest that IBPs can potentiate PRE activity by stimulating pairing-sensitive silencing (PSS) and bringing PREs on each homologous chromosome into close proximity [14]. Further studies on IBP-regulated LRIs will provide a better understanding regarding the involvement of insulator proteins in the implementation of cellular genetic programs.

Here we investigate the role of the Suppressor of Hairy wings (Su(Hw)) IBP in the formation of specific LRIs. We demonstrate that Su(Hw) is capable of forming LRIs with Combgap, a protein involved in the recruitment of PcG group proteins to chromatin [15, 16]. By analysing wild-type and Su(Hw) loss-of-function *Drosophila* ovaries, we examine the genomic context and functional output of these LRIs.

## Results

### Su(Hw) enables the indirect binding of Combgap to chromatin

While loss of Su(Hw) binding to chromatin is not lethal for flies, it does lead to female sterility through a mid-stage arrest of oogenesis and egg chamber degeneration at approximately stage 9 [17, 18]. Thus, *Drosophila* ovaries are the most biologically relevant organ to study Su(Hw) functioning. In this study, we used *su(Hw)*^*v/E8*^ heteroallelic mutant flies (henceforth referred to as Su(Hw) loss of function or Su(Hw)^LOF^ flies), which completely lack Su(Hw) binding to chromatin due to a point mutation in the *su(Hw)* coding region for zinc finger 7 [18]. Female flies carrying *su(Hw)*^*v/E8*^ alleles are sterile and experience egg chamber degeneration at stage 9 [17, 18]. To compare wild type and Su(Hw)^LOF^ ovaries, we dissected ovaries from recently eclosed 15-hour-old flies that contain egg chambers no older than stage 8 (following the procedure in [19]).

**Fig. 1:**
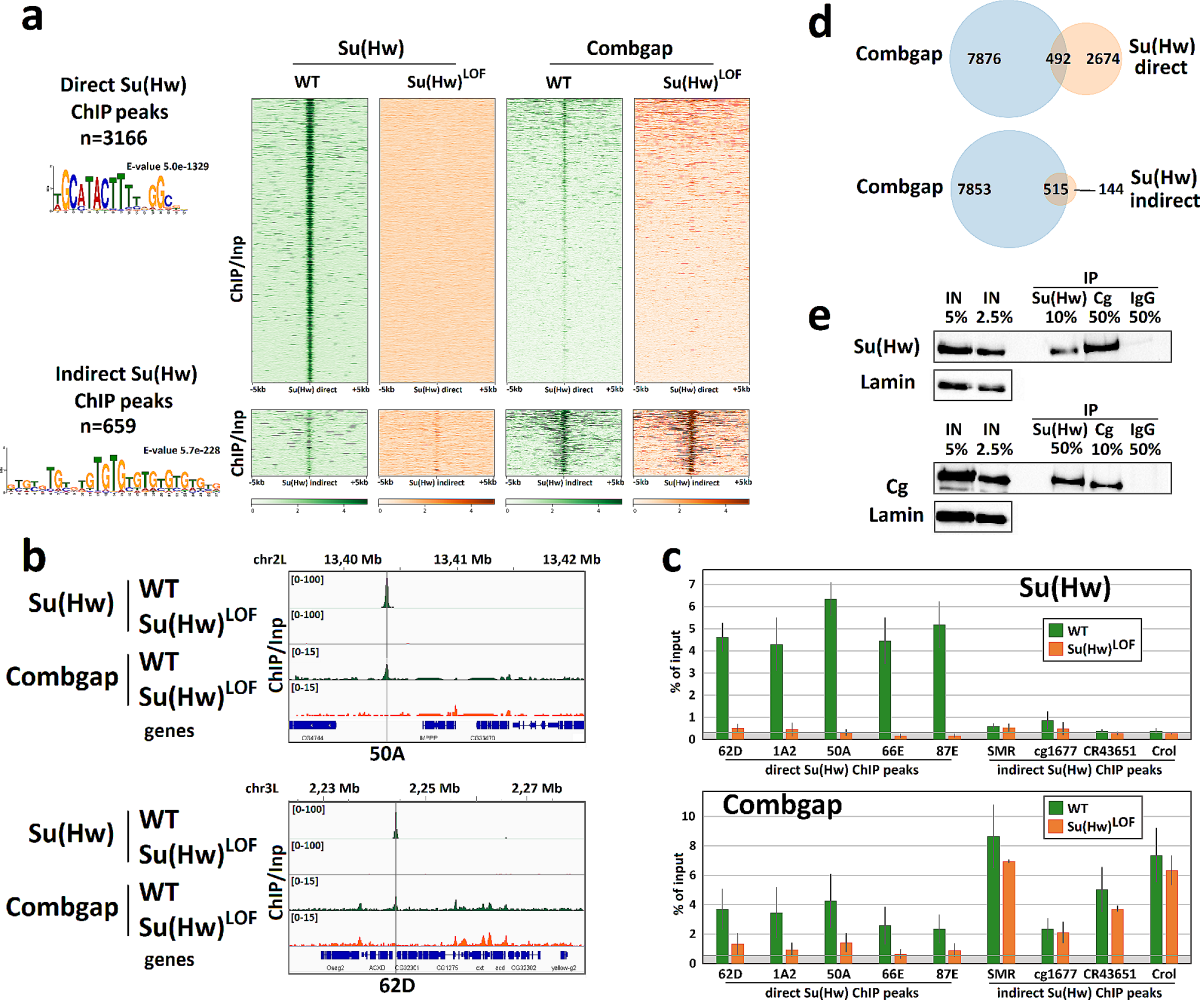
Combgap binds Su(Hw) ChIP-Seq peaks in Su(Hw)-dependant manner. **(a)** Heatmaps of Su(Hw) and Combgap ChIP/Inp signal on Su(Hw) ChIP-Seq peaks with direct and indirect Su(Hw) binding. Heatmaps are made for the wild type (WT, green) and Su(Hw)^LOF^ (orange) *Drosophila* ovaries and are sorted by the strength of the median Su(Hw) ChIP/Inp signal in the wild type *Drosophila* ovaries. The DNA-motifs determined by MEME suite 5.4.1 [20, 55] are shown on the left from each group of peaks. **(b)** Genome browser (IGV) examples highlighting that Combgap binding to 62D and 50A insulators is Su(Hw)-dependent. **(c)** ChIP analysis of Su(Hw) and Combgap binding to direct and indirect Su(Hw) ChIP-Seq peaks in the wild type (green columns) and Su(Hw)^LOF^ (orange columns) ovaries assessed by qRT-PCR. Well-known Su(Hw)-dependent insulators (62D, 1A2, 50A, 66E, 87E) were used as direct Su(Hw) ChIP-Seq peaks. The Y-axis represents the % of input chromatin fraction. The gray area on the diagrams indicates the Su(Hw) and Combgap binding levels on 1A1 negative control region in the wild type ovaries. The data are mean values from three independent experiments, error bars represent standard deviations. **(d)** Intersection of Combgap ChIP-Seq peaks with direct and indirect Su(Hw) ChIP-Seq peaks in the wild type *Drosophila* ovaries. **(e)** Immunoprecipitations (IPs) from nuclear protein extracts of *Drosophila* S2 cells. IPs were performed with antibodies against Combgap (Cg) and Su(Hw) (the total purified IgG antibodies of non-immunized rabbits (IgG) were used as a negative control), which is indicated on the top of the figure. Western blots were stained with the antibodies indicated on the left of the figure. Anti-lamin staining was used as a loading control for input protein extracts. All input and IP samples were loaded on a single western blot. The original Western blots are present in Supplementary Fig. 10. The numbers above the inputs and IPs represent a portion of a loaded fraction (in respect to the amount used for IPs)

In our previous study [19], we had shown that Su(Hw) ChIP-Seq peaks in the ovaries included a cluster of peaks that lacked the Su(Hw) DNA-binding motif (henceforth referred to as as indirect Su(Hw) peaks). Instead, these indirect Su(Hw) peaks contained a GTGT-motif, previously associated with the binding of Combgap, a protein involved in the recruitment of PcG group proteins [15, 16]. Here, we confirmed the binding of Combgap to these indirect Su(Hw) peaks both in wild-type and Su(Hw)^LOF^ ovaries using ChIP-Seq with antibodies against Combgap (Fig. 1a-c). The majority of indirect Su(Hw) peaks are included in the set of Combgap peaks: 78% of indirect Su(Hw) peaks (515 out of 659 peaks) intersect with Combgap peaks (Fig. 1d). Additionally, we observed that Combgap also binds to a portion of direct Su(Hw) peaks (492 peaks out of 3166 direct Su(Hw) peaks), and this binding is Su(Hw)-dependent (Fig. 1a-b, Supplementary Fig. 1a). Using ChIP experiments coupled with qPCR, we confirmed the binding of Combgap to direct and indirect Su(Hw) ChIP peaks (Fig. 1c). We selected the 100 Su(Hw) peaks with the strongest Combgap binding (direct Su(Hw) peaks) and analysed the DNA motifs present in this set using the MEME-ChIP program [20]. We found a strong enrichment only for the Su(Hw) DNA motif, not the Combgap DNA motif, indicating a preference for Su(Hw)-mediated Combgap recruitment to these sites (Supplementary Fig. 1b).

To validate the ChIP-Seq findings, we conducted co-immunoprecipitation experiments (co-IPs) in nuclear protein extract from *Drosophila* S2 cells to test an interaction between Su(Hw) and Combgap. Antibodies against Su(Hw) successfully co-precipitated Combgap from the nuclear extract and vice versa (Fig. 1e). Moreover, recent immunoaffinity purification of the Combgap interactome coupled with high throughput mass spectrometry (IP/LC-MS) revealed statistically significant enrichment of Su(Hw) and its partners Mod(mdg4) and CP190, providing further support for the existence of protein-protein interaction between Su(Hw) and Combgap [21].

### Su(Hw)-bound Combgap is associated with active chromatin rather that polycomb-directed repression

Originally, Combgap and Su(Hw) were characterized as proteins linked to repressed chromatin: Combgap has been identified as a protein capable of recruiting the Ph subunit of the PRC1 transcription repression complex to chromatin [15] and approximately 86% of Su(Hw) ChIP peaks were observed within repressed chromatin regions in the S2 cell line [22, 23]. However, subsequent research has shown that both Combgap and Su(Hw) also interact with proteins associated with active chromatin: Combgap ChIP-Seq peaks have been found to colocalise with RNA polymerase II pausing factors and the transcription start sites (TSSs) of active genes [21] while Su(Hw) has been found to co-immunoprecipitate with several transcription coactivators [19, 23]. This prompted us to investigate the chromatin features associated with Su(Hw)-Combgap interactions.

**Fig. 2:**
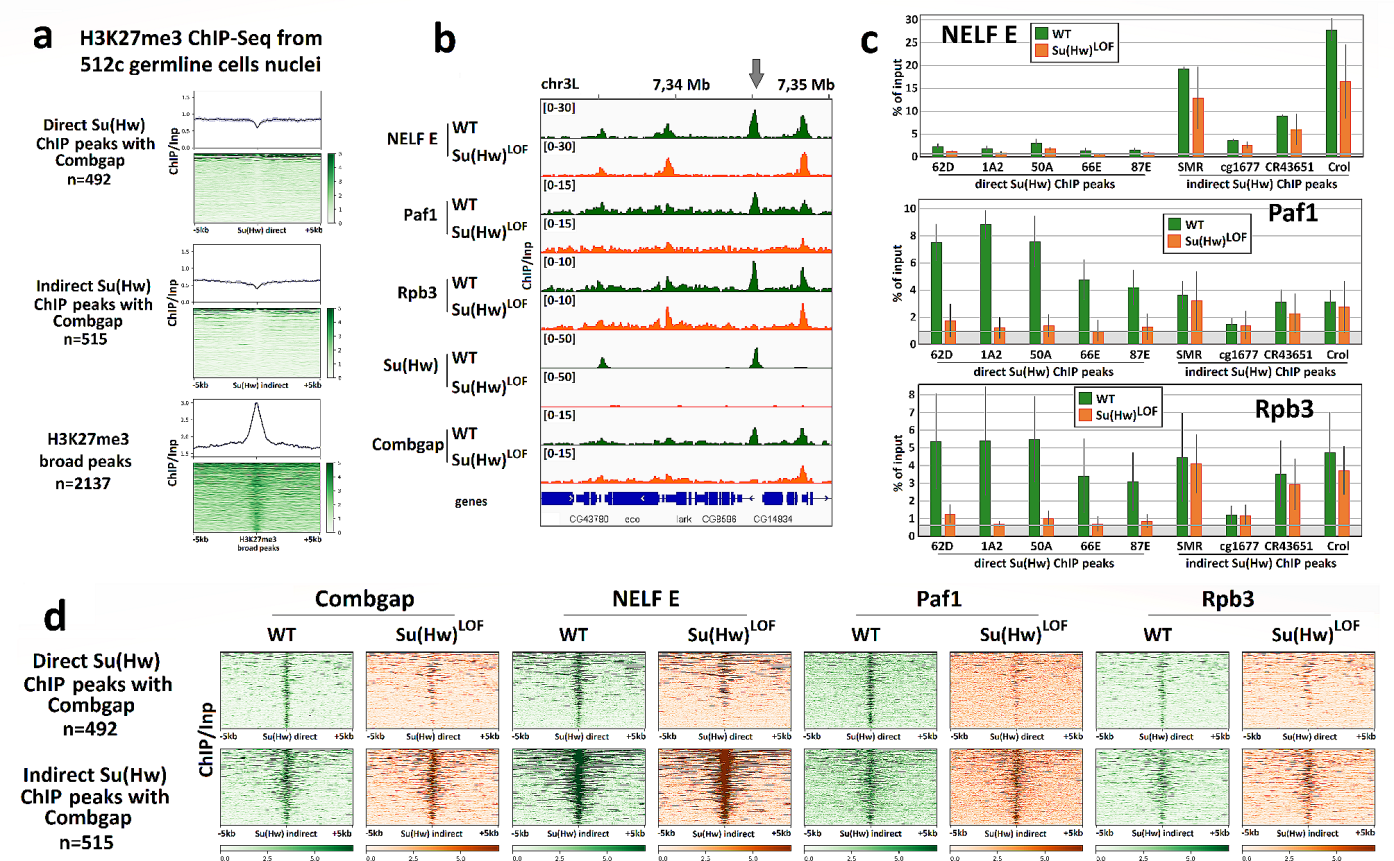
Su(Hw)-bound Combgap is associated with active chromatin factors. **(a)** Heatmaps and pile-up profiles of H3K27me3 ChIP/Inp signal from 512c FACS-sorted germline cells nuclei [24] on direct and indirect ovarian Su(Hw) ChIP peaks, intersecting with Combgap, and H3K27me3 broad peaks. Heatmaps are sorted by the strength of the median H3K27me3 ChIP/Inp signal. **(b)** Genome browser (IGV) example highlighting that NELF E, Paf1 and Rpb3 binding to direct Su(Hw) ChIP-Seq peaks coincides with Combgap binding and is Su(Hw)-dependent. The position of direct Su(Hw) ChIP-Seq peak is marked on top of the tracks with an arrow. **(c)** ChIP analysis of NELF E, Paf1 and Rpb3 binding to direct and indirect Su(Hw) ChIP-Seq peaks in the wild type (green columns) and Su(Hw)^LOF^ (orange columns) ovaries assessed by qRT-PCR. Well-known Su(Hw)-dependent insulators (62D, 1A2, 50A, 66E, 87E) were used as direct Su(Hw) ChIP-Seq peaks. The Y-axis represents the % of input chromatin fraction. The gray area on the diagrams indicates the NELF E, Paf1 and Rpb3 binding levels on 1A1 negative control region in the wild type ovaries. The data are mean values from three independent experiments, error bars represent standard deviations. **(d)** Heatmaps of Combgap, NELF E subunit of NELF complex, Paf1 positive elongation factor and Rpb3 RNAPII subunit ChIP/Inp signals on direct Su(Hw) ChIP-Seq peaks, intersecting with Combgap, and on indirect Su(Hw) ChIP-Seq peaks, intersecting with Combgap. Heatmaps are made for the wild type (WT, green) and Su(Hw)^LOF^ (orange) *Drosophila* ovaries and are sorted by the strength of the median NELF E ChIP/Inp signal in the wild type *Drosophila* ovaries

To determine whether the H3K27me3 Polycomb-dependent chromatin mark is associated with Su(Hw)-Combgap interactions in *Drosophila* ovary we utilised the H3K27me3 ChIP-Seq from 512c germline cells nuclei, FACS-sorted from the ovaries of 3–6 days old females of *Drosophila melanogaster* [24]. We examined the distribution of H3K27me3 on 492 direct Su(Hw) ChIP peaks that intersect with Combgap, 515 indirect Su(Hw) ChIP peaks that intersect with Combgap, and on H3K27me3 broad domains, annotated from this H3K27me3 ChIP-Seq (Fig. 2a). Results show no H3K27me3 enrichment, neither on direct nor indirect Su(Hw) peaks with Combgap, compared to H3K27me3 broad domains. Hence, the Su(Hw)-Combgap interaction does not appear to be associated with Polycomb-mediated heterochromatin establishment.

To investigate the potential link between Su(Hw)-Combgap interactions and RNAP II pausing factors, we conducted ChIP-Seq experiments on wild-type and Su(Hw)^LOF^ ovaries using antibodies against the NELF E subunit of NELF, the Paf1 positive elongation factor, and the Rpb3 RNAP II subunit. We found significant Su(Hw)-dependent enrichment of NELF E, Paf1, and Rpb3 at direct Su(Hw) peaks containing Combgap (Fig. 2b-d, Supplementary Fig. 2a). The binding of NELF E, Paf1 and Rpb3 to direct Su(Hw) ChIP peaks was further verified through ChIP experiments coupled with qPCR (Fig. 2c). Notably, direct Su(Hw) peaks bound to NELF E (326 peaks), Paf1 (398 peaks), and Rpb3 (277 peaks) in wild-type *Drosophila* ovaries account for only 10.3%, 12.6%, and 8.7% of all direct Su(Hw) peaks (3166 peaks), respectively. On the other hand, 85% of direct Su(Hw)-NELF E, 80% of direct Su(Hw)-Paf1, and 94% of direct Su(Hw)-Rpb3 peaks also intersect with Combgap peaks (Supplementary Fig. 2b). These observations suggest that the binding of RNA polymerase II and pausing factors to direct Su(Hw) peaks is related to Combgap rather than being intrinsic to Su(Hw).

### The majority of Su(Hw)^LOF^ down-regulated promoters are located within 2 kb of Combgap peaks

**Fig. 3:**
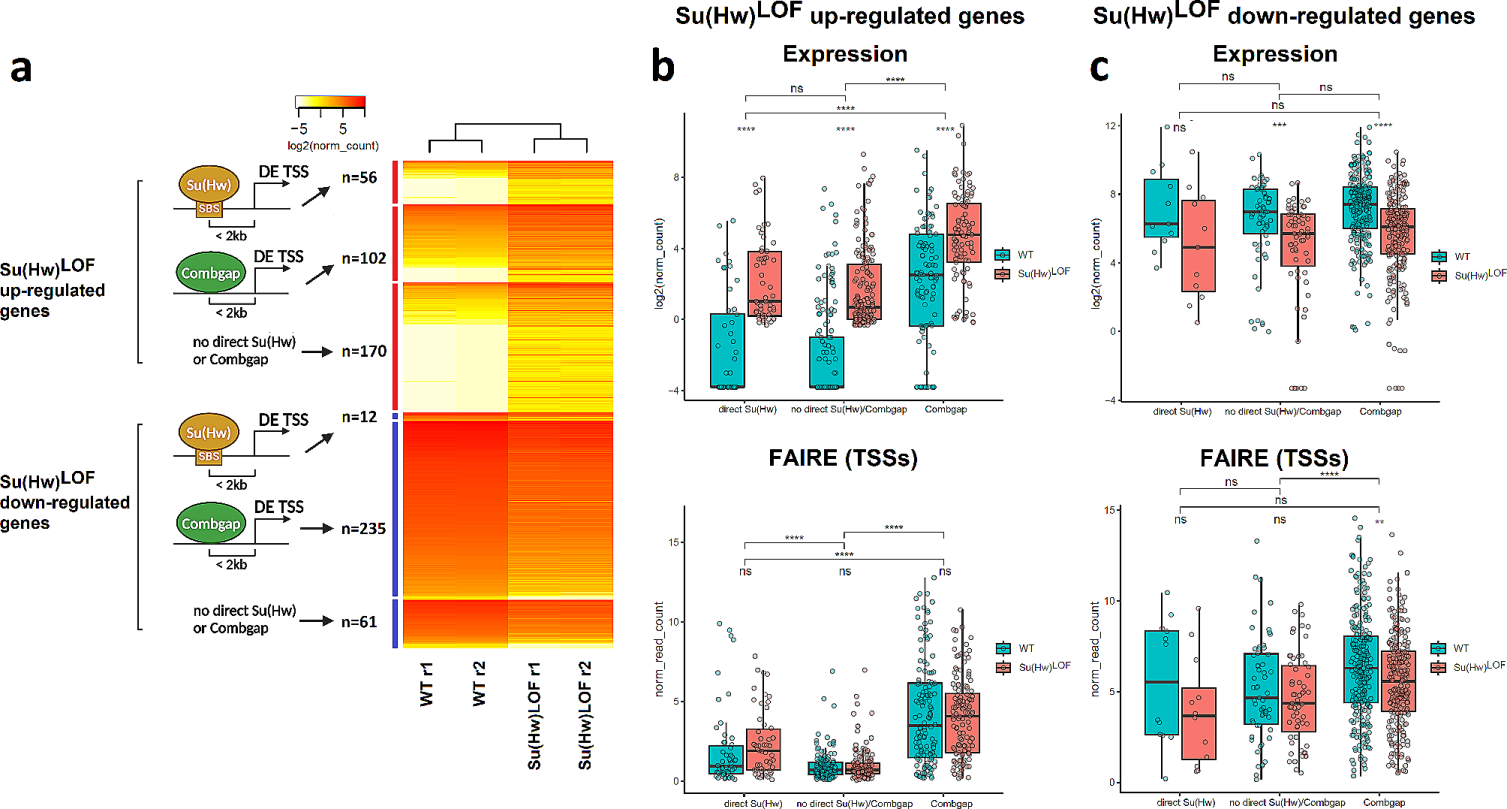
The Su(Hw)^LOF^ mis-regulated TSSs are often located within 2 kb of Combgap peaks and possess distinct chromatin properties. **(a)** Heatmap of the genes, differentially expressed in Su(Hw)^LOF^ ovaries (logFC > 1, adjusted *P*-value < 0.01). Up- and down-regulated genes are clustered in 3 categories: (1) with direct Su(Hw) peak within 2 kb from TSSs (these peaks contain directly bound Su(Hw) with and without Combgap) (2), with Combgap peaks within 2 kb from TSSs (excluding those which already appeared in the first group - these peaks contain Combgap with and without indirectly bound Su(Hw), but do not contain directly bound Su(Hw)), and (3) without direct Su(Hw)/Combgap peaks near TSSs. The log_2_ of normalised read counts are shown. **(b)** and **(c)** The box plots showing main statistical features of the expression and of the chromatin state (according to FAIRE signal on TSSs) for Su(Hw)^LOF^ up- and down-regulated genes, correspondingly. Within each box plot, the thick line in the centre indicates median expression; boxes and whiskers around it represent 25–75 percentile interval and minimum/maximum expression values, respectively. The dots on the plots represent the features of individual genes. ****, *** and ** are for adjusted *P*-values of < 0.0001, < 0.001 and < 0.01, correspondingly, according to T-test

It is well-known that disruption of Su(Hw) binding to chromatin leads to abnormal transcription in *Drosophila* ovaries [25, 26]. However, the underlying mechanism that mediates the impact of Su(Hw) on gene transcription remains unknown. To investigate whether the interactions between Su(Hw) and Combgap are associated with transcriptional regulation, we first identified all differentially expressed (DE) genes in Su(Hw)^LOF^ ovaries compared with wild-type ovaries. We purified mRNA from the corresponding ovaries and performed RNA sequencing (RNA-Seq) on the cDNA libraries obtained (RNA-Seq was performed in two biological replicates for each genotype). We identified 636 transcripts that were significantly DE in Su(Hw)^LOF^ mutants compared to WT (with adjusted *p*-value < 0.01 and fold-change > 2) (Supplementary Fig. 3a-b, Supplementary Table 1, Fig. 3a).

Previous studies have reported that 35% of genes mis-regulated in *su(Hw)*^*-/-*^ mutant ovaries have Su(Hw) peaks located inside or within 2 kb upstream or downstream of these genes [25]. Using the same criteria, we found that 34.4% (218) of DE transcripts were located within 2 kb of direct Su(Hw) ChIP-Seq peaks. We also compared the proportion of DE TSSs that intersect with direct Su(Hw) and Combgap ChIP-Seq peaks by separately analysing Su(Hw)^LOF^ up- and down-regulated TSSs (Fig. 3a). We observed that 17.1% (56 out of 328) of Su(Hw)^LOF^ up-regulated TSSs were located within 2 kb of direct Su(Hw) peaks, whereas only 3.9% (12 out of 308) of Su(Hw)^LOF^ down-regulated TSSs had direct Su(Hw) peaks within a distance of 2 kb (these direct Su(Hw) peaks contained directly bound Su(Hw) with and without Combgap). Surprisingly, 76.3% (235 out of 308) of Su(Hw)^LOF^ down-regulated TSSs had Combgap ChIP-Seq peaks (these peaks contained Combgap with and without indirectly bound Su(Hw), but did not contain directly bound Su(Hw)) within 2 kb, while only 31.1% (102 out of 328) of Su(Hw)^LOF^ up-regulated TSSs and 41.2% of all *D. melanogaster* TSSs localised within 2 kb of Combgap ChIP-Seq peaks. This suggests that Combgap may be involved in the transcription regulation of Su(Hw)^LOF^ down-regulated TSSs. Interestingly, the median expression levels of Su(Hw)^LOF^ down-regulated genes with TSSs within 2 kb of direct Su(Hw) and Combgap ChIP-Seq peaks were similar, while those of Su(Hw)^LOF^ up-regulated genes varied significantly (Fig. 3b-c). Su(Hw)^LOF^ up-regulated genes containing direct Su(Hw) peaks within 2 kb showed notably lower median expression compared with Su(Hw)^LOF^ up-regulated genes whose TSSs colocalise with Combgap.

To further clarify the chromatin features of genes that are mis-regulated in Su(Hw)^LOF^ ovaries, we analysed MNase-Seq data for follicular cells from egg chamber stages 1–8 [27] and FAIRE-Seq data for wild-type and Su(Hw)^LOF^ ovaries [19]. Among Su(Hw)^LOF^ down-regulated TSSs, TSSs with Combgap within 2 kb according to FAIRE-Seq typically had the most open chromatin state, which decreased in Su(Hw)^LOF^ compared to WT and exhibited visible nucleosome phasing (Fig. 3c, Supplementary Fig. 3d). Su(Hw)^LOF^ up-regulated TSSs with Combgap within 2 kb also exhibited the highest chromatin accessibility among other downregulated TSSs (Fig. 3b). In contrast, Su(Hw)^LOF^ up-regulated genes with direct Su(Hw) peaks within 2 kb of their TSSs, exhibited features of genes that are repressed or inactive in wild-type ovaries, in general (Fig. 3b, Supplementary Fig. 3d). This suggests that this group of genes reflects the earlier described property of Su(Hw) to repress transcription [25].

We further analysed the DNA motifs enriched in Combgap ChIP peaks, flanking DE TSS (Fig. 3a), to test whether they contain the Combgap DNA-binding motif. Interestingly, the Combgap ChIP-Seq peaks located within 2 kb of Su(Hw)^LOF^ down-regulated TSSs were enriched with BEAF32 and M1BP DNA motifs, in addition to Combgap’s own DNA-binding motifs (Supplementary Fig. 3e). Previous studies have shown that both M1BP and BEAF32 flank promoters [28, 29] and active chromatin domains [8], hence the enrichment of their motifs aligns with our observation that Su(Hw)^LOF^ down-regulated TSSs are characterised by more open chromatin then Su(Hw)^LOF^ up-regulated TSSs (Fig. 3B-C). For Combgap ChIP-Seq peaks located within 2 kb of Su(Hw)^LOF^ up-regulated TSSs we found enrichment of Combgap and GAF DNA motifs. Taken together, these observations suggest that, in addition to Combgap and Su(Hw), other DNA binding proteins may be involved in the regulation of Su(Hw)^LOF^ DE genes.

### Su(Hw) ChIP-Seq peaks form long-range chromatin interactions with Combgap ChIP-Seq peaks

A previous study has shown that the absence of the BEAF32 DNA-binding motif in BEAF32 IBP ChIP-Seq peaks indicates the presence of LRIs between chromatin-bound BEAF32 and its partner proteins [30]. To determine whether similar LRIs exist between Su(Hw) and Combgap ChIP-Seq peaks, we conducted in situ Hi-C on ovaries dissected from wild-type and Su(Hw)^LOF^ flies using the four-cutter restriction enzyme MboI. Two biological replicates were conducted for each genotype. The Hi-C data obtained totalled 266–276 million raw pair-end reads per genotype with 66–70 million of valid Hi-C pairs per genotype (the statistics for the read alignments, mapped reads and valid Hi-C pairs can be found in Supplementary Fig. 4 and Supplementary Table 2).

**Fig. 4:**
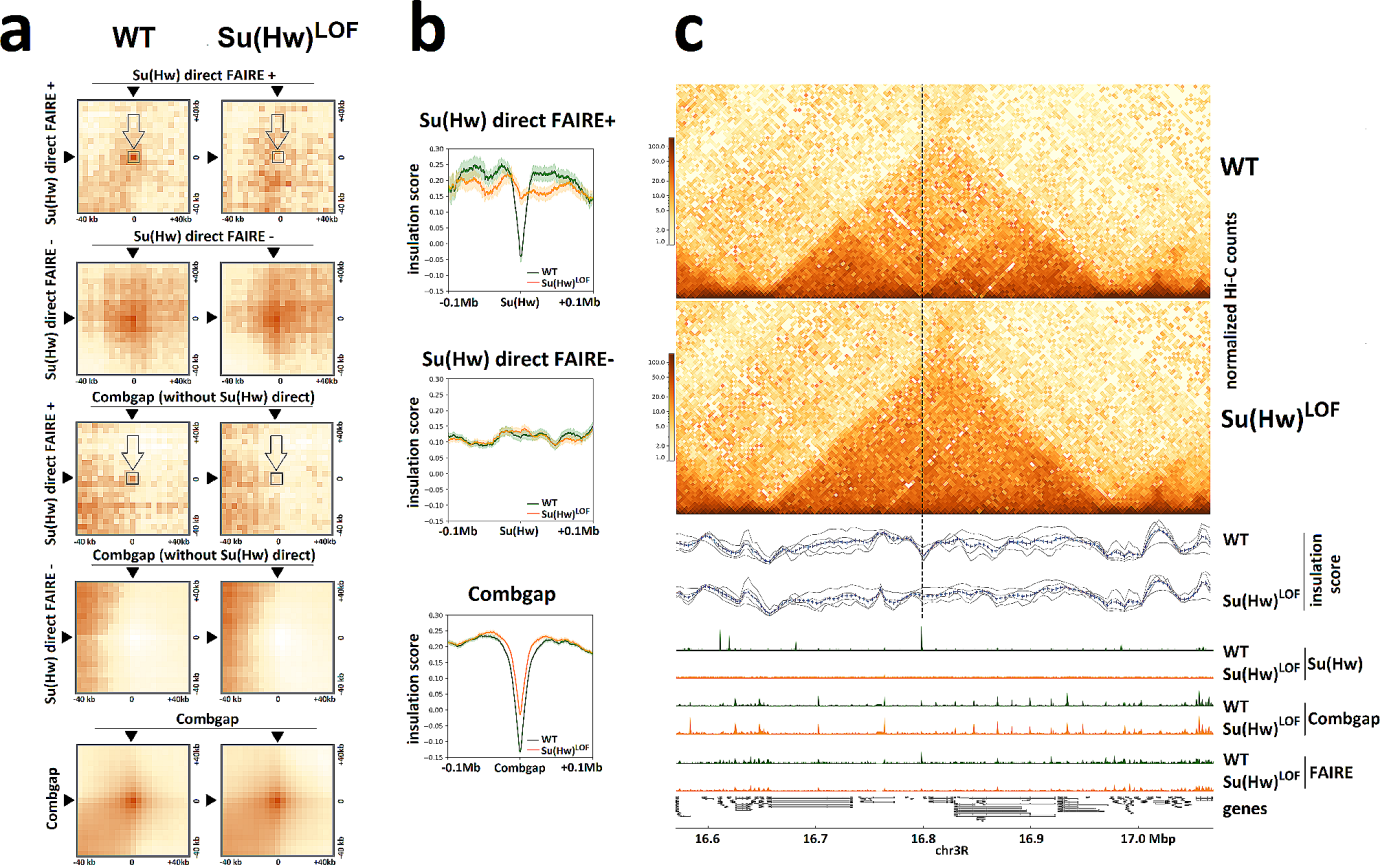
Long-range interactions between Su(Hw) and Combgap ChIP-Seq peaks. **(a)** Averaged spatial interactions between the different sets of Su(Hw) and Combgap ChIP-Seq peaks in the wild-type (WT) and Su(Hw)^LOF^ ovaries, estimated with a coolpup.py program [31]. The minimal and maximal distances of interactions were set at 200 kb and 1000 kb, correspondingly, the pad size is ± 40 kb around the central pixel. **(b)** Profile plots of insulation score at direct Su(Hw) FAIRE+, Su(Hw) FAIRE- and Combgap ChIP-Seq peaks in the wild-type (WT) and Su(Hw)^LOF^ ovaries. The pile-up profiles were generated as a median of insulation score signal. The standard error is displayed on the profiles as semi-transparent area around the main line of the profiles. **(c)** Hi-C matrices from the wild-type (WT) and Su(Hw)^LOF^ ovaries, showing one genomic region with insulator scores and occupancies of Su(Hw), Combgap (ChIP-Seq) and open chromatin regions (according to FAIRE-Seq) in the wild-type (WT) and Su(Hw)^LOF^ ovaries. The image was generated using pyGenomeTracks [60]. This particular region was selected to illustrate Su(Hw)-dependence of long range interactions (LRIs) between direct Su(Hw) FAIRE + and Combgap ChIP-Seq peaks (position of direct Su(Hw) FAIRE + peak in these LRIs is marked with the vertical line)

We used the coolpup.py program to estimate the average LRIs between different pairs of ChIP-Seq peak [31]. As a positive control, we examined the average spatial interactions between Rpb3 (a subunit of RNAPII) peaks and found a strong enrichment of Rpb3-Rpb3 LRIs (Supplementary Fig. 5a). We also observed significant enrichment of Combgap-Combgap LRIs (Fig. 4a). This is consistent with previous finding that Combgap can recruit the Ph subunit of the PRC1 complex, which is often found at the anchors of chromatin loops in *Drosophila* [15, 32]. Importantly, both Rpb3-Rpb3 and Combgap-Combgap LRIs remained intact in Su(Hw)^LOF^ flies, indicating that these LRIs are independent of Su(Hw). Next, we estimated the average spatial interactions within the set of direct Su(Hw) ChIP-Seq peaks. Although we found clear enrichment of LRIs between direct Su(Hw) ChIP-Seq peaks, we did not detect any differences in these LRIs in the Su(Hw)^LOF^ background (Supplementary Fig. 5a).

In a previous study, we demonstrated that direct Su(Hw) ChIP-Seq peaks in *Drosophila* ovaries can be classified into two clusters based on their enrichment with the FAIRE signal and active chromatin features, such as chromatin remodelers and Gcn5 histone acetyltransferase (henceforth referred to as Su(Hw) direct FAIRE + peaks), or lack of such enrichment (Su(Hw) direct FAIRE- peaks) [19]. We analysed the average LRIs for these two clusters separately. We observed the LRIs between Su(Hw) direct FAIRE + peaks disappeared in the absence of Su(Hw) (Fig. 4a). Interestingly, for Su(Hw) direct FAIRE- peaks, the LRIs appeared to be independent of Su(Hw) binding to chromatin. When we analysed the average insulation profiles for these groups of peaks, we observed that Su(Hw) direct FAIRE + peaks showed strong insulation scores (Fig. 4b). In Su(Hw)^LOF^, the insulation score for Su(Hw) direct FAIRE + peaks decreased, indicating that Su(Hw) can define local insulation at the domain borders where it binds. On the other hand, Su(Hw) direct FAIRE- peaks did not show any enrichment in insulation score, suggesting that they do not colocalise with the domain borders.

To investigate the spatial interactions between Combgap and Su(Hw), we excluded all peaks from the Combgap peak set that intersected with direct Su(Hw) ChIP-Seq peaks. Results showed the presence of LRIs between Combgap and Su(Hw) direct FAIRE + peaks, but not with Su(Hw) direct FAIRE- peaks (Fig. 4a). In Su(Hw)^LOF^ flies, these LRIs disappeared, indicating the importance of Su(Hw) for their formation. These Su(Hw)-dependent LRIs between Su(Hw) direct FAIRE + peaks and Combgap are also evident in Hi-C maps (Fig. 4c, Supplementary Fig. 5c-g).

We also investigated the interactions between Su(Hw) direct FAIRE + and FAIRE- peaks and the ChIP peaks of M1BP and BEAF32 proteins, for which we detected motifs in the Combgap ChIP peaks within 2 kb of Su(Hw)^LOF^ down-regulated TSSs (Supplementary Fig. 3e). We observed that similarly to the interaction with Combgap, Su(Hw) direct FAIRE + peaks show Su(Hw)-dependent LRIs with M1BP and BEAF32 (Supplementary Fig. 5b). This observation demonstrates that other architectural proteins, such as M1BP and BEAF32, may also be involved in the establishment and maintenance of Su(Hw)-dependent LRIs.

To validate the findings of our Hi-C experiments, we examined the existing Hi-C datasets [33] for the OSC cell line, which has been derived from somatic cells of the *Drosophila* ovary. Results showed long-range interactions in the Hi-C data from OSC cells, similar to our observations in wild-type ovaries (Supplementary Fig. 6).

### Changes in chromatin architecture in Su(Hw)^LOF^ background correlate with transcription mis-regulation

To investigate whether changes in chromatin architecture correlate with transcriptional mis-regulation in Su(Hw)^LOF^ ovaries, we analysed the average spatial interaction between Su(Hw) direct FAIRE + peaks and Combgap localised within 2 kb of Su(Hw)^LOF^ DE genes (Supplementary Fig. 7). For distances ranging from 50 to 200 kb, we did not observe any significant interactions between these regions, likely due to the shorter average ranges of transcriptional regulatory interactions (such as enhancer-promoter interactions). Indeed, it was shown that the majority of characterised enhancers are within 10 kb of their target gene, with only a few capable of acting at distances beyond 50 kb [34]. Unfortunately, the analysis of interactions at distances below 50 kb is not possible with the obtained Hi-C matrices due to the high basal level of contacts at such short distances.

**Fig. 5:**
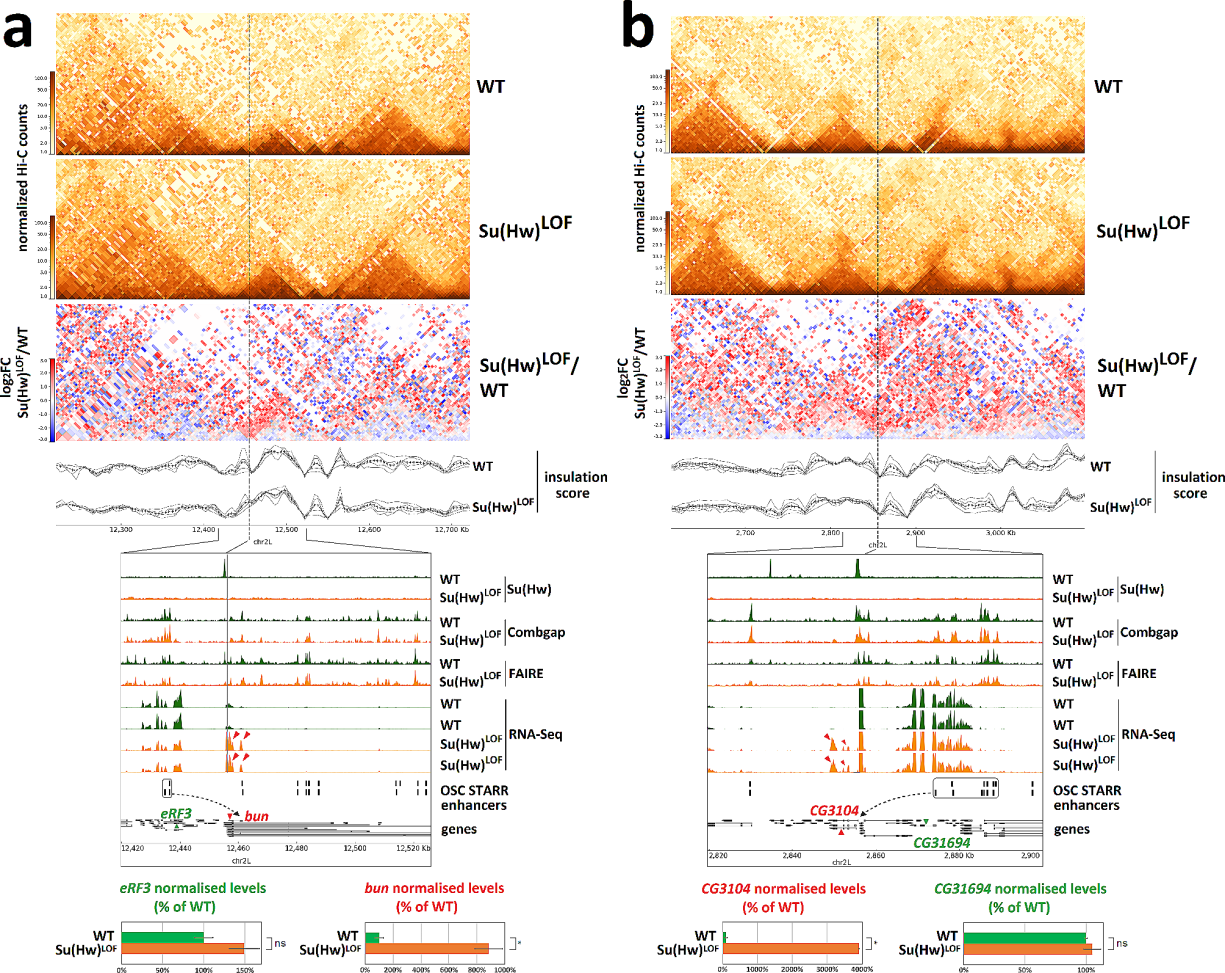
LRIs between Su(Hw) and Combgap ChIP-Seq peaks correlate with transcription mis-regulation in Su(Hw)^LOF^ background. **(a-b)** Two loci displaying enhancer hijacking. Top of the figure: Hi-C and differential Hi-C matrices (log_2_FC) of the *bun* (**a**) and *CG3104* (**b**) loci. Bottom of the figure: zoom-in of Su(Hw), Combgap ChIP-Seq and FAIRE-Seq in WT (green) and Su(Hw)^LOF^ (orange) ovaries and RNA-Seq separately for 2 replicates from WT (green) and Su(Hw)^LOF^ (orange) ovaries. Known OSC STARR enhancers are shown; Su(Hw)-dependent boundaries weakened in Su(Hw)^LOF^ are marked with grey dashed lines; potential newly formed enhancer-promotor interactions are shown in dotted arrows. Genes highlighted in red are up-regulated in Su(Hw)^LOF^ (*bun, CG3104*), while the possible enhancer’s original targets (*eRF3, CG31694*) are in green. RNA-Seq normalized levels are displayed in bar plots; n.s. is for adjusted *P*-value > 0.05 and * is for adjusted *P*-value < 0.05

Analysing Hi-C maps for wild-type and Su(Hw)^LOF^ ovaries, we observed that the disruption of Su(Hw)-Combgap LRIs correlated with the transcriptional mis-regulation of nearby genes in a Su(Hw)^LOF^ background (Supplementary Fig. 8). It is already known that changes in LRIs can affect enhancer-promoter communication within interacting domains [6, 7]. In the OSC cell line, derived from somatic ovary cells, all DNA elements capable of acting as enhancers have been annotated [35]. Following the approach from Cavalheiro et al. [36] to analyse enhancer hijacking, we observed that Su(Hw)-Combgap boundaries, which are disrupted in Su(Hw)^LOF^, often localise near “hijacked” OSC enhancers (Fig. 5). This suggests that changes in Su(Hw)-Combgap LRIs may impact the enhancer-promoter interaction networks of DE genes in the somatic cells of the ovary.

To identify changes in the binding of transcriptional regulators to mis-regulated genes in Su(Hw)^LOF^, we analysed the ChIP-Seq distribution of Combgap, chromatin remodelers [19], the Rpb3 subunit of RNA polymerase II, the NELF E subunit of NELF complex, and the Paf1 positive elongation factor binding along mis-regulated genes (Supplementary Fig. 9). These transcriptional regulators have been shown to bind Su(Hw) ChIP peaks in Su(Hw)-dependent manner [19]. We found no significant changes in the distribution of these proteins along Su(Hw)^LOF^ up-regulated genes (Supplementary Fig. 9b). However, for Su(Hw)^LOF^ down-regulated genes that have Combgap located within 2 kb of their TSSs, we observed an increase in the binding of Combgap, Rpb3, and ISWI ATPase of the ISWI chromatin remodeler family, along with a decrease in the binding of Brm ATPase of the SWI/SNF family and CHD1 ATPase of the CHD family (Supplementary Fig. 9a). For Su(Hw)^LOF^ down-regulated genes that have direct Su(Hw) peaks within 2 kb of their TSSs, we observed a substantial decrease in the binding of Brm ATPase and Mi2 ATPase of the CHD chromatin remodeler family, along with a reduction in Paf1 binding (Supplementary Fig. 9a). Previous studies have shown that ISWI and Mi-2, in addition to their role in transcription activation [37, 38], can contribute to the establishment of an inactive chromatin state [39–42], while Brm and CHD1 are mainly involved in chromatin opening resulting in transcription activation [43, 44]. The increased ISWI binding at Su(Hw)^LOF^ down-regulated genes on a Su(Hw)^LOF^ background suggests that ISWI can act as a co-repressor of this group of genes, while decreased Brm, Mi2 and CHD1 levels correlate with the role of these remodelers as transcriptional co-activators.

## Discussion

**Fig. 6:**
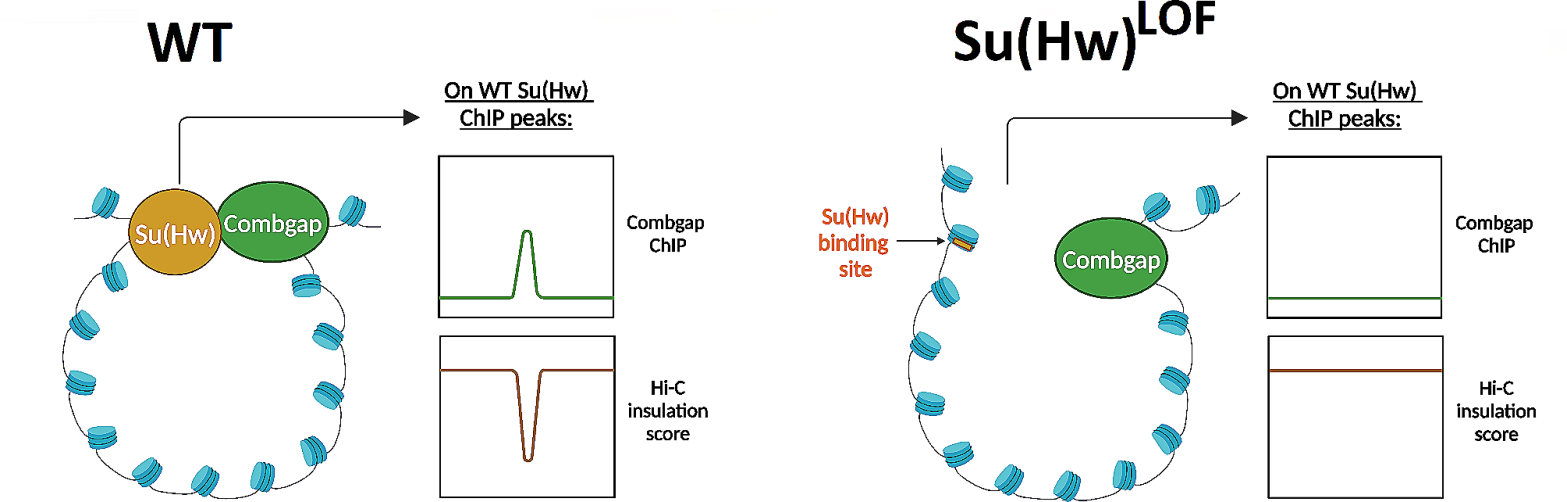
Model of the long-range interactions between direct Su(Hw) FAIRE + and Combgap ChIP-Seq peaks

Our study provides the first direct evidence of the involvement of Su(Hw) IBP in maintaining chromatin architecture in *Drosophila* ovaries. Consistent with previous research on BEAF32 IBP indirect chromatin binding [9], we could show that the indirect binding of Su(Hw) to chromatin serves as an indication of Su(Hw) participation in long-range chromatin interactions. Previously, we had identified a cluster of indirect Su(Hw) ChIP-Seq peaks in *Drosophila* ovaries that contained a GTGT-motif instead of the Su(Hw) DNA-binding motif [19]. Here, we found that these indirect Su(Hw) peaks were occupied by Combgap, a protein known to recruit PcG group proteins to chromatin [15, 16]. Moreover, we could show that Combgap also binds to a subset of direct Su(Hw) peaks in a Su(Hw)-dependent manner. Our in situ Hi-C experiments revealed the presence of Su(Hw)-dependent long-range chromatin interactions between Combgap and a portion of the direct Su(Hw) ChIP-Seq peaks (Fig. 6).

We suggest that Centrosomal Protein 190 kD (CP190), a well-known cofactor of various *Drosophila* insulators and mediator of interactions between different IBPs, may play a role in sustaining the observed Su(Hw)-Combgap interactions. In support of this hypothesis, a previous study has found a statistically significant enrichment of CP190 in Combgap immunoprecipitation followed by mass spectrometry analysis (IP/LC-MS) [21]. To further investigate the role played by CP190 in Su(Hw)-Combgap interactions, additional immunoprecipitation experiments conducted on the background of CP190 depletion are needed.

Intriguingly, Combgap-bound Su(Hw) peaks exhibit features typically found in active rather than inactive chromatin regions. However, on average, the majority of Su(Hw) peaks colocalise within repressed chromatin types [25, 45] (Fig. 2a-c, Supplementary Fig. 2a). We believe that the presence of active chromatin factors such as RNA polymerase II, pausing factors, and chromatin remodelers [19, 23, 45] in direct Su(Hw) ChIP-Seq peaks is directly linked to the long-range interactions between Combgap and Su(Hw). Additionally, our observations may indicate a potential involvement of Combgap in non-repressive functions of Polycomb [46, 47]. However, the mechanisms underlying this possibility remain elusive, which highlights the need for further research in this area. In particular, Combgap depletion from the ovary will elucidate its role in the recruitment of active chromatin factors to Su(Hw) sites.

Our in situ Hi-C experiments revealed that direct Su(Hw) sites exhibit heterogeneity in their ability to support long-range interactions (Fig. 4a, b; Supplementary Fig. 5a). Previous DNA-motif analysis of Su(Hw) binding sites together with the analysis of available ChIP-Seq data has shown that Su(Hw) binding sites can be categorised into distinct groups, based on their intersection with active chromatin features [45]. Our ChIP-Seq data from wild-type and Su(Hw)^LOF^*Drosophila* ovaries confirmed this observation. We showed that direct Su(Hw) ChIP-Seq peaks can be classified into two clusters: those enriched with FAIRE signal and active chromatin proteins (Su(Hw) direct FAIRE + peaks), and those lacking these features (Su(Hw) direct FAIRE- peaks) [19]. In situ Hi-C experiments demonstrated that both Su(Hw)-dependent LRIs and domain border insulation are only characteristic of Su(Hw) direct FAIRE + peaks (Fig. 4a, b). Su(Hw) direct FAIRE- peaks do not colocalise with domain boundaries and retain the ability to self-interact even in the absence of Su(Hw). We hypothesise that Su(Hw) direct FAIRE- peaks retain the ability to self-interact in the absence of Su(Hw) due to other proteins maintaining the interactions between these chromatin regions. This interpretation is supported by previous studies which have shown that, while Su(Hw) is enriched at the borders and inside lamina-associated domains (LADs), knockdown of Su(Hw) only causes minimal changes in the interactions of these chromatin regions with the nuclear lamina [48], i.e. LADs can retain their self-interaction in a Su(Hw)^LOF^ background. Our data suggest that the involvement of Su(Hw) in transcriptional regulation [25, 26], particularly its role in activating the transcription of certain genes, may be related to its ability to establish long-range interactions. Su(Hw)^LOF^ up-regulated genes, containing direct Su(Hw) peaks within 2 kb of their TSSs, have closed chromatin at their promoters, do not bind transcriptional regulators, and have low median transcription level in wild-type *Drosophila* ovaries (Fig. 3b, Supplementary Fig. 9b). We believe that these genes are a direct reflection of the ability of Su(Hw) to repress transcription [25, 26]. On the other hand, Su(Hw)^LOF^ down-regulated genes, containing Combgap or direct Su(Hw) peaks within 2 kb of their promoters, and Su(Hw)^LOF^ up-regulated genes with Combgap within 2 kb of their TSSs have open chromatin on TSSs and Su(Hw)-dependent binding of some transcriptional regulators (Fig. 3, Supplementary Fig. 9). We suggest that the long-range interactions between Combgap and direct Su(Hw) peaks are involved in the formation of the local environment regulating the transcription of Su(Hw)^LOF^ mis-regulated genes (Fig. 5, Supplementary Fig. 8, Fig. 6).

## Conclusions

Our study demonstrates that Su(Hw) insulator binding protein can form long-range interactions with Combgap, Polycomb response elements binding protein, and that these interactions are associated with active chromatin factors rather than with Polycomb dependent repression.

## Methods

### Collection of the *Drosophila* ovaries

The flies of Oregon-R-modENCODE (the wild-type control, corresponds to Bloomington stock 25211) and *su(Hw)*^*v/E8*^ (a kind gift of A. Golovnin laboratory) stocks were used. All flies were raised at 25 °C on standard agar medium. Ovaries were dissected from recently eclosed 15 h-old flies (contain only egg chamber stages 1–8) the same way as in [19]. During dissection, we thoroughly controlled the correctness of the stages of egg chambers in the ovaries, collected for the analysis.

### Nuclear protein extract and immunoprecipitation

*Drosophila* Schneider cell line 2 (S2) cells were used for nuclear protein extract preparation. S2 cells were maintained at 25 °C in Schneider’s insect medium (Sigma) containing 10% FBS (HyClone). The S2 cells nuclear protein extract was obtained as described previously [49]. Immunoprecipitations were performed as previously described [49].

### ChIP, ChIP-Seq, ChIP-Seq analysis

For the ChIP experiments ovaries were collected in PBS buffer (50 pairs per ChIP), cross-linked with 1% of formaldehyde for 5 min and then incubated for 5 min with 125 mM Glycine. After that, ovaries were washed with PBS buffer for three times. The remaining ChIP protocol was performed as described previously [23, 50]. The primers used for qPCR analysis of ChIP experiments are listed in Supplementary Table 3.

ChIP-Seq libraries were obtained using the NEBNext Ultra^™^ II DNA library preparation kit (New England Biolabs). Only the library fragments of 250–500 bp were subjected to next generation sequencing (NGS). NGS was performed by Evrogen (evrogen.ru) and Genetico (genetico.ru) on the Illumina NovaSeq6000 sequencer. For each of the ChIP-Seq libraries approximately 4–12 millions of unique single-end reads were obtained. The single‐end reads in FastQ format were mapped to the *Drosophila* genome assembly dm6 using Bowtie2 [51] (Galaxy Version 2.3.4.3) and filtered (with minimum MAPQ quality score = 5).

BigWig files were generated using bamCoverage [52] (Galaxy Version 3.5.1.0.0) with scores representing number of reads per kilobase per million (RPKM). The final BigWig files (representing the protein-binding profiles) were obtained using bigwigcompare [52] (Galaxy Version 3.5.1.0.0) as a ratio of ChIP signal to Input. The peaks of proteins’ binding were defined by MACS2 callpeak [53] (Galaxy Version 2.1.1.20160309.6) with the following parameters: -gsize ‘120,000,000’ -keep-dup ‘1’-qvalue ‘0.01’ -mfold ‘5’ ‘50’ --bw ‘350’ 2 > &1 > macs2_stderr. Corresponding input DNA was used as a control for peak calling.

Visualization of ChIP-Seq data in the heatmaps and pile-up profiles was performed on the Galaxy-P platform [54]. All ChIP-Seq data obtained in the current study were deposited into the Gene Expression Omnibus, GSE231576.

The definition of DNA motifs for different ChIP-Seq peaks was performed with MEME suite 5.4.1 [20, 55]. The motifs were searched in the regions flanking the ChIP-Seq summit by 250 bp.

The intersection of the MACS2-called ChIP-Seq peaks was performed using the regions flanking the ChIP-Seq summit by 150 bp, with the bedtools Intersect intervals [56] (Galaxy Version 2.30.0 + galaxy1).

For the ovaries of egg chamber stages 1–8 we used the following the ChIP-Seq and MNase-Seq data previously described: ChIP-Seq for Su(Hw), Brm, ISWI, Mi2, CHD1 [19] (GSE168894), H3K27me3 ChIP-Seq from 512c germline cells nuclei, FACS-sorted from the ovaries of 3–6 days old *Drosophila* females [24] and MNase-Seq data for follicular cells from egg chambers stages 1–8 [27] (NCBI-SRA BioProject SRP057811). We have not used any figures or text from the manuscripts previously published, only analysed the data deposited at the free public access databases.

As Polycomb response elements (PREs) for S2 cell line we used the same set of regions as in [21].

### RNA-Seq, RNA-Seq analysis

For extraction of RNA the ovaries of 15 h old wild-type (*oregon*) and *su(Hw)*^*v/E8*^ females were collected in PBS buffer (50 pairs per sample), in two biological repeats. Total RNA was extracted with the TRI reagent (Ambion). PolyA comprising RNA fraction was isolated and prepared for sequencing with the NEBNext Ultra^™^ II Directional RNA Library Prep Kit. NGS was performed by Evrogen (evrogen.ru) with the Illumina NovaSeq6000 sequencer. For each RNA-Seq library approximately 15–32 millions of unique single‐end reads were obtained. Obtained reads were mapped to the *Drosophila* genome assembly dm6 using HISAT2 [57] (Galaxy Version 2.2.1 + galaxy0). BigWig files were generated using bamCoverage [52] (Galaxy Version 3.3.2.0.0) with scores representing number of reads per kilobase per million (RPKM). Differential analysis was employed by limma [58] (Galaxy Version 3.48.0 + galaxy1). Obtained RNA-Seq data were deposited into the Gene Expression Omnibus, GSE231576.

### In situ Hi-C, Hi-C analysis

For the in situ Hi-C experiments, ovaries were collected in PBS buffer (200 pairs per Hi-C), cross-linked with 1% of formaldehyde for 5 min and then incubated for 5 min with 125 mM Glycine. Then ovaries were washed with 1xPBS buffer for three times. The remaining in situ Hi-C protocol was performed as described previously [59]. In situ Hi-C was performed in two biological replicates per each genotype. Hi-C libraries were obtained using the NEBNext Ultra^™^ II DNA library preparation kit (New England Biolabs). Only the library fragments of 200–600 bp were subjected to NGS sequencing. NGS was performed by Genetico (genetico.ru) on the Illumina NovaSeq6000 sequencer. For each genotype in total 266–276 million of raw paired-end reads were obtained (the statistics for the read alignments, mapped reads and valid Hi-C pairs generated in hicBuildMatrix 3.4.2 for raw contact matrices can be found in Supplementary Fig. 4 and Supplementary Table 2).

Hi-C data was processed using the HiCExplorer suite version 3.4.2 [60]. The paired-end reads in FastQ format were separately mapped to the *Drosophila* genome assembly dm6 using Bowtie2 [51] (Galaxy Version 2.4.2 + galaxy0), with the following options: --sensitive-local --reorder. The raw contact Hi-C matrices in .h5 format with the bins size of 4 kb were generated in hicBuildMatrix [60] (Galaxy Version 3.4.2.0). The percentage of mapped reads, self-ligation, same-fragment, self-circle and duplicates for the generated in hicBuildMatrix raw contact matrices is presented on Supplementary Fig. 4. The biological replicates of the raw contact Hi-C matrices were pooled for each genotype. The resulting raw contact matrices were normalized using hicCorrectMatrix (Imakaev’s iterative correction), after running hicCorrectMatrix in diagnostic mode to extract optimal minimal and maximal values for the filterThreshold option (we removed the lowest 1% and the highest 0.05% of interchromosomal contacts to avoid vastly underrepresented or overrepresents regions). Insulator scores were obtained on normalized matrices with 4 kb bin sized using hicFindTADs with q-value 0.01 cut-off. Following the recommendations of the authors of the program, we set --minDepth 12000 –maxDepth 40000 and --step 4000 for the 4 kb binned matrices. To generate genomic regions views we used pyGenomeTracks 3.5.1.

Averaged spatial interactions between different sets of ChIP-Seq peaks were calculated using coolpup.py program v0.9.5 [31]. The minimal and maximal distances of interactions were set at 200 kb and 1000 kb, correspondingly (except for the average spatial interactions on Supplementary Fig. 7, for this we used 50–200 kb distance range). The pad size was set at ± 40 kb or ± 24 kb around the central pixel and 10 randomly shifted control regions were used to normalise the signal for local background.

The following previously described Hi-C datasets for the OSC *Drosophila* cells, treated with EGFP dsRNA [33], were used: GSM4790413, GSM4790414, GSM4790421, GSM4790422, GSM4790427, GSM4790428 from GSE158082. The following FASTQ datasets were combined together and processed the same way as described above, except for the 6 kb bin size used to generate the matrix and the following setting of hicFindTADs: --minDepth 18000 --maxDepth 60000 and --step 6000.

### Antibodies

For the co-immunoprecipitations and ChIP-Seq experiments we used previously described antibodies against Combgap [21], Su(Hw) [19], M1BP [29], BEAF32 [61], Paf1, NELF E and Rpb3 [62].

### Electronic supplementary material

Below is the link to the electronic supplementary material.

Supplementary Material 1

## Data Availability

Obtained sequencing data are deposited into the Gene Expression Omnibus, GSE231576.

## Abbreviations

IBP: Insulator–binding protein
TAD: Topologically–associated domain
LRE: Long range interaction
DE: Differentially expressed
TSS: Transcription start site
