# Supplementary material for: Su(Hw) interacts with Combgap to establish long-range chromatin contacts
Source: Epigenetics Chromatin. 2024 May 21;17:17. doi: 10.1186/s13072-024-00541-x (PMC11106861; doi:10.1186/s13072-024-00541-x)
Supplement: Supplementary file 1 — Supplementary Material 1 [file 13072_2024_541_MOESM1_ESM.docx]

**Su(Hw) interacts with Combgap to establish long-range chromatin contacts**

Nadezhda E. Vorobyeva^1,2^, Alexey N. Krasnov^1^, Maksim Erokhin^1^, Darya Chetverina^1^, Marina Mazina^1,2^*

^1^ Institute of Gene Biology, Russian Academy of Sciences, Moscow 119334, Russia

^2^ Center for Precision Genome Editing and Genetic Technologies for Biomedicine, Institute of Gene Biology, Russian Academy of Sciences

* Corresponding author (email to mazinam@genebiology.ru)


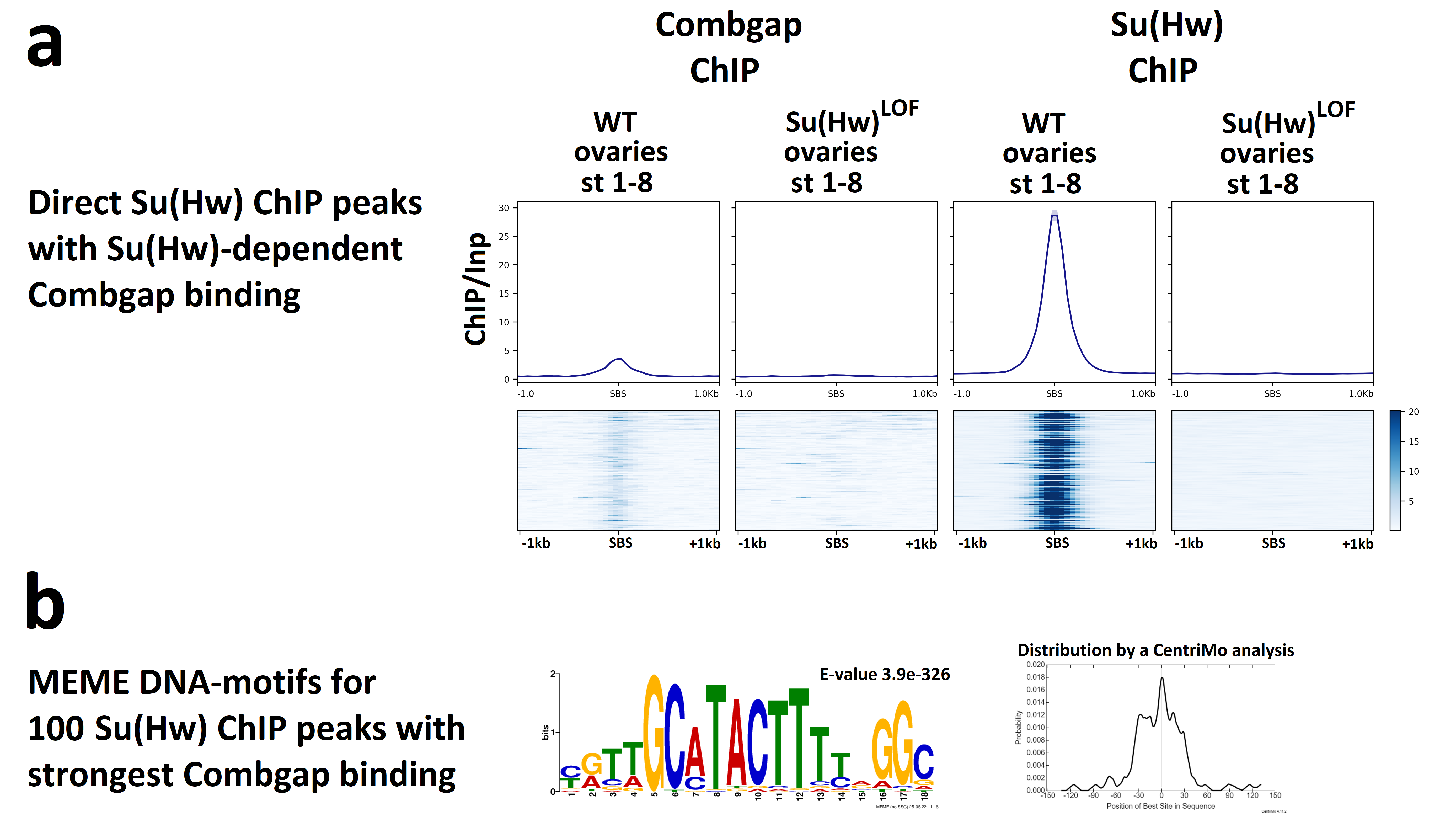


**Supplementary figure 1. Su(Hw) enables Combgap binding to Su(Hw) ChIP-Seq peaks.**

**(a)** Heatmaps of Combgap and Su(Hw) ChIP/Inp signal on direct Su(Hw) ChIP-Seq peaks intersecting with Combgap ChIP-Seq peaks. Heatmaps are made for the wild type (WT) and Su(Hw)^LOF^ *Drosophila* ovaries and are sorted by the strength of the median Su(Hw) ChIP/Inp signal in the wild type *Drosophila* ovaries.

**(b)** The DNA-motifs determined by MEME suite 5.4.1 in the set of 100 Su(Hw) ChIP-Seq peaks with the strongest Combgap binding.


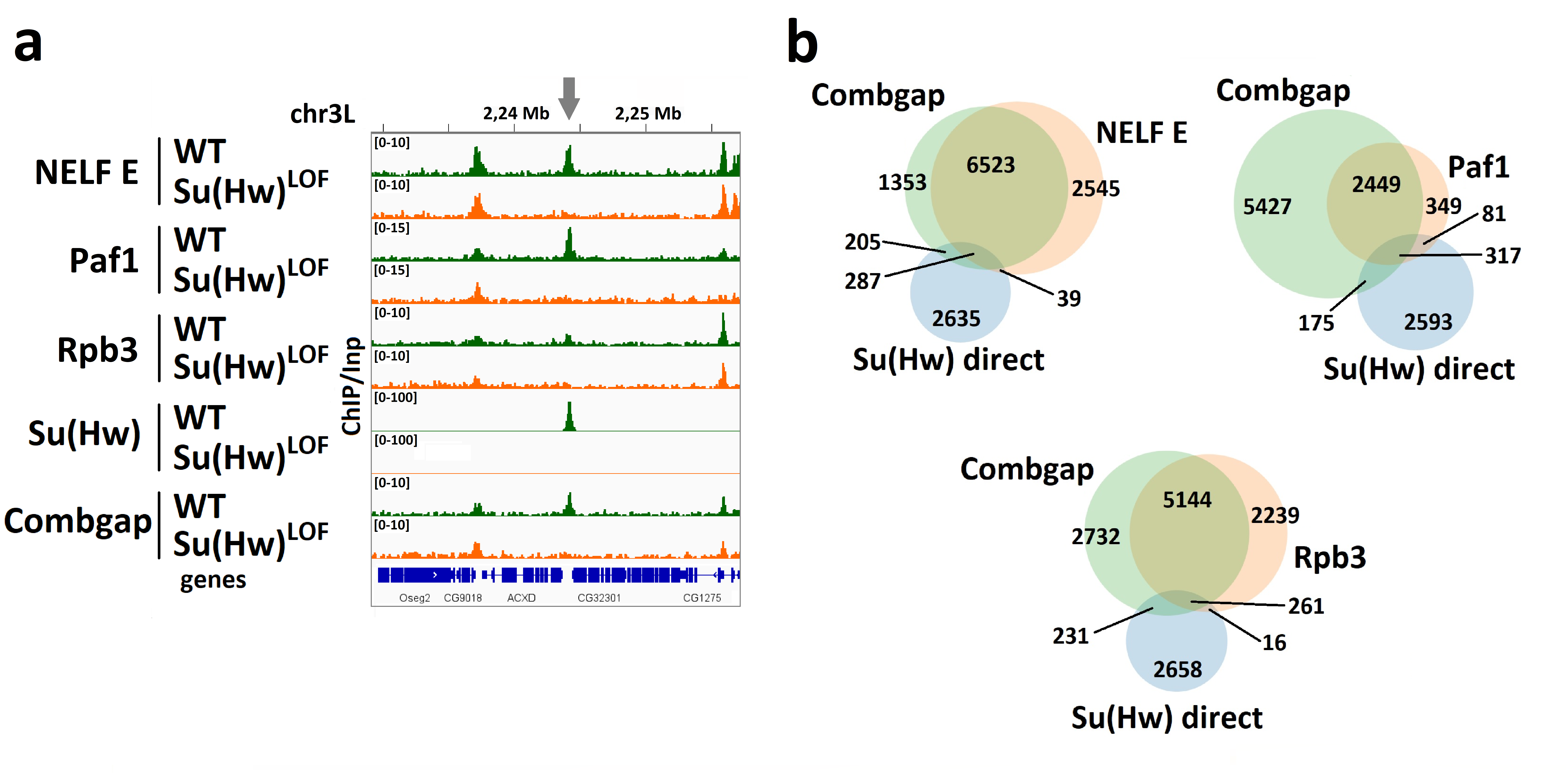


**Supplementary figure 2. Common Su(Hw)-Combgap ChIP-Seq peaks are associated with active chromatin factors rather that Polycomb-directed repression.**

**(a)** Genome browser (IGV) example highlighting that NELF E, Paf1 and Rpb3 binding to direct Su(Hw) ChIP-Seq peaks coincides with Combgap binding and is Su(Hw)-dependent. The position of direct Su(Hw) ChIP-Seq peak is marked on top of the tracks with an arrow.

**(b)** Colocalisation of direct Su(Hw) ChIP-Seq peaks with Combgap, NELF E, Paf1 and Rpb3 ChIP-Seq peaks in the wild type *Drosophila* ovaries


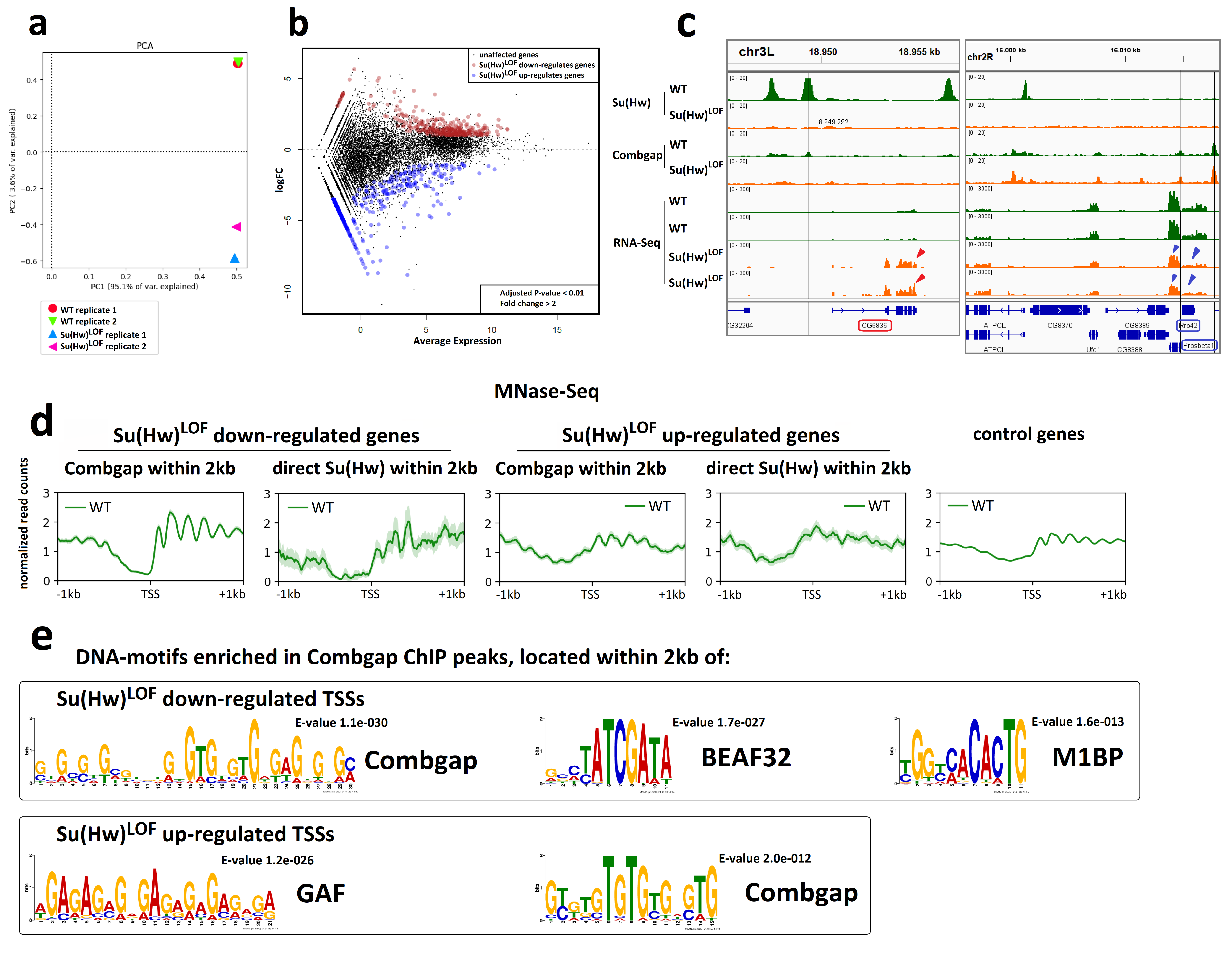


**Supplementary figure 3. Characteristics of Su(Hw)^LOF^ mis-regulated genes.**

**(a)** PCA correlation plot for two biological replicates of RNA-Seq experiments from the wild-type (WT) and Su(Hw)^LOF^ ovaries of egg chamber stages 1-8.

**(b)** The MD-plot showing the changes in gene expression (according to RNA-Seq data) upon Su(Hw)^LOF^ compared to the wild-type (WT) ovaries of egg chamber stages 1-8. Significantly down- and up-regulated genes are highlighted in red and blue, respectively.

**(c)** Genome browser (IGV) example highlighting Su(Hw)^LOF^ mis-regulated genes with direct Su(Hw) peak or Combgap peak within 2 kb from TSSs. The positions of mis-regulated genes are marked with arrows.

**(d)** Average profiles of MNase-Seq from follicular cells from egg chamber stages 1–8 on the TSSs, up- and down-regulated in Su(Hw)^LOF^, correspondingly. The pile-up profiles were generated for the mis-regulated and control TSSs. As a set of control TSSs we used TSSs, expression of which does not change significantly in Su(Hw)^LOF^ ovaries compared to the wild-type (fold-change < 2). The standard error is displayed on the profiles as semi-transparent area around the main line of the profiles.

**(e)** DNA-binding motifs identified by MEME suite 5.4.1 (Machanick and Bailey (2011) Bioinformatics 27, 1696-1697) in Combgap ChIP-Seq peaks, located within 2 kbp of Su(Hw)^LOF^ up- and down-regulated genes.


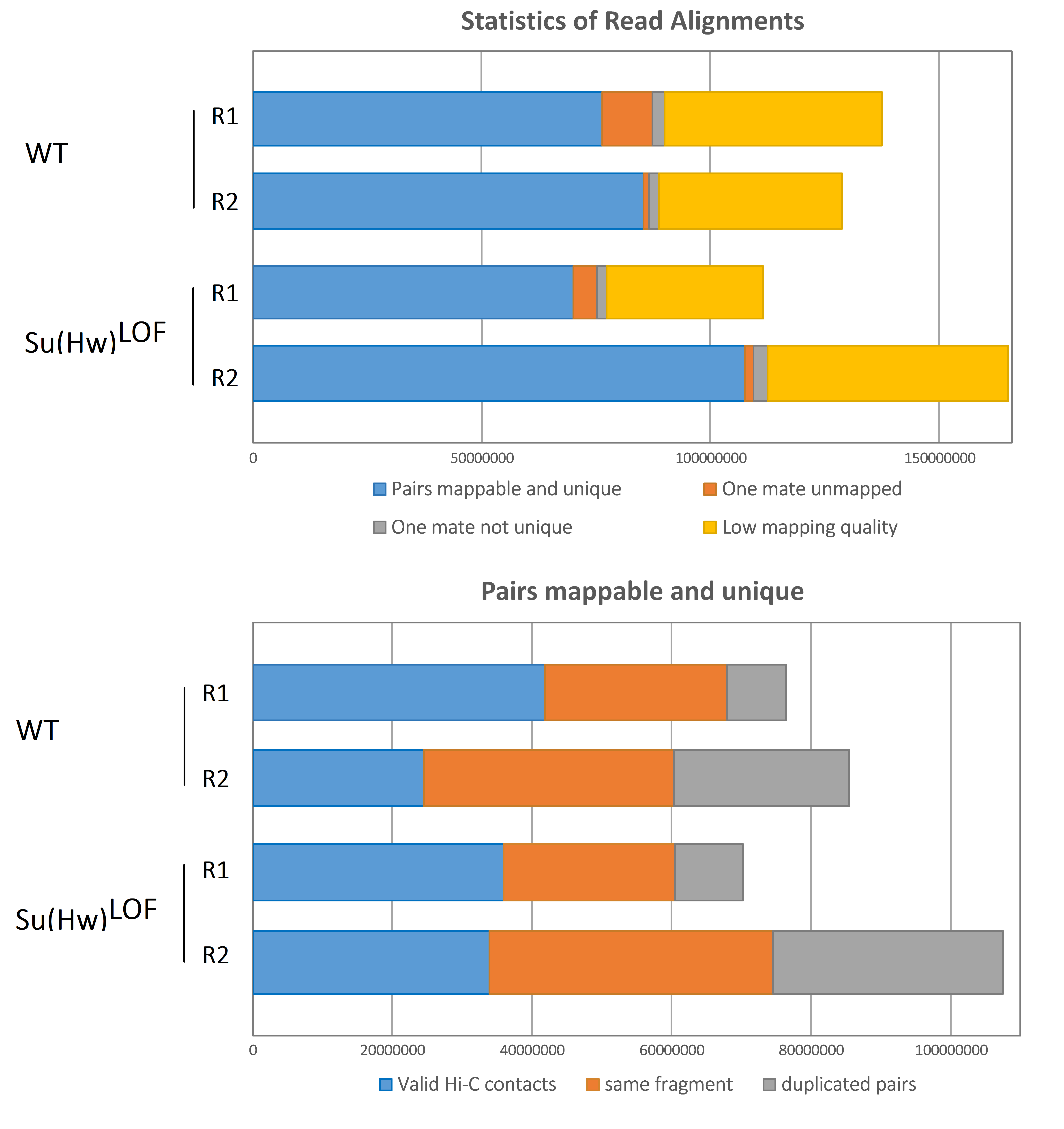


**Supplementary figure 4. The statistics for Hi-C data, obtained in the study.**

The read alignments, mapped reads and valid Hi-C pairs are generated in hicBuildMatrix 3.4.2 for raw contact matrices in the wild-type (WT) and Su(Hw)^LOF^ ovaries of egg chamber stages 1-8 (also present in Supplementary Table 2).


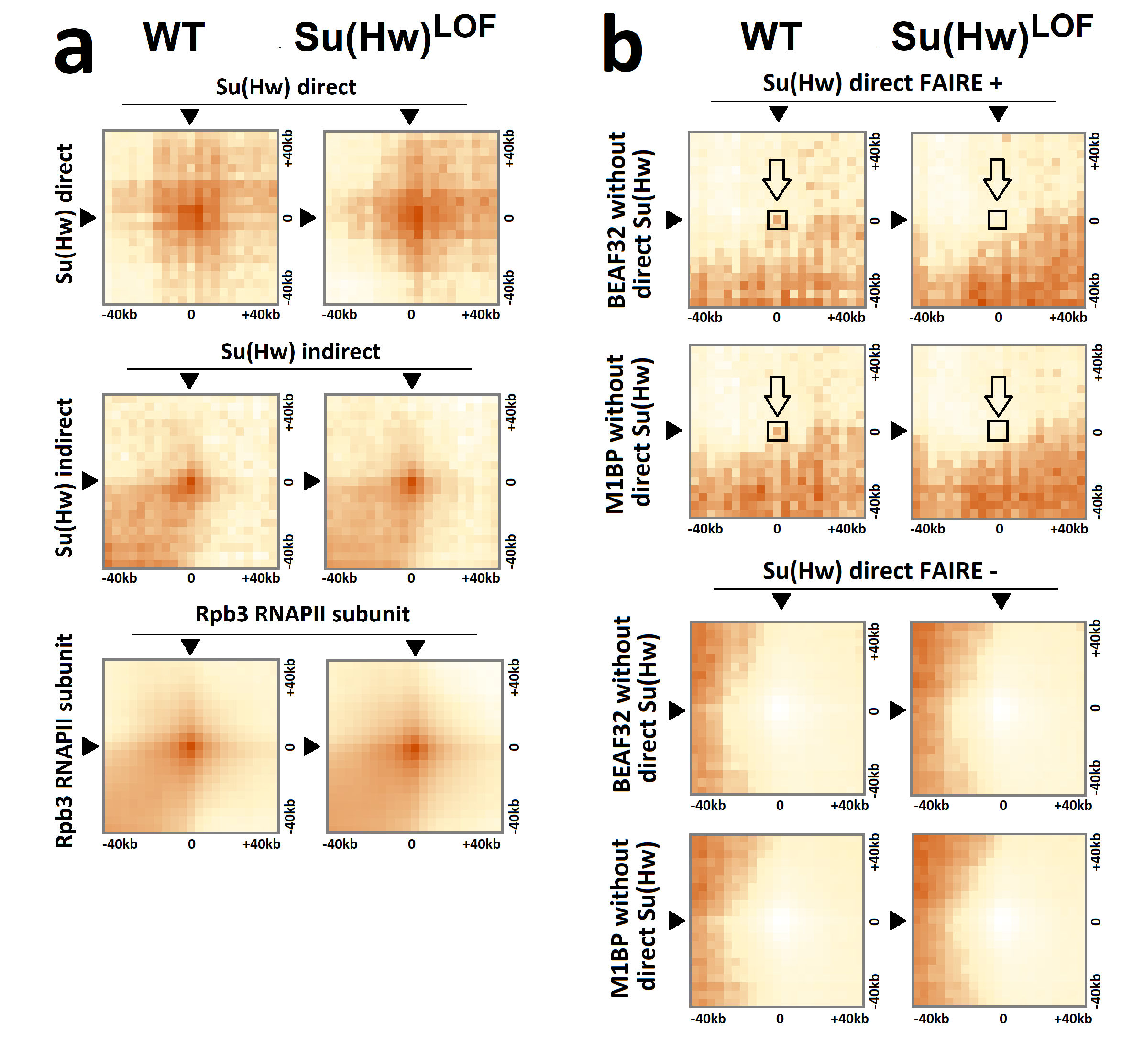


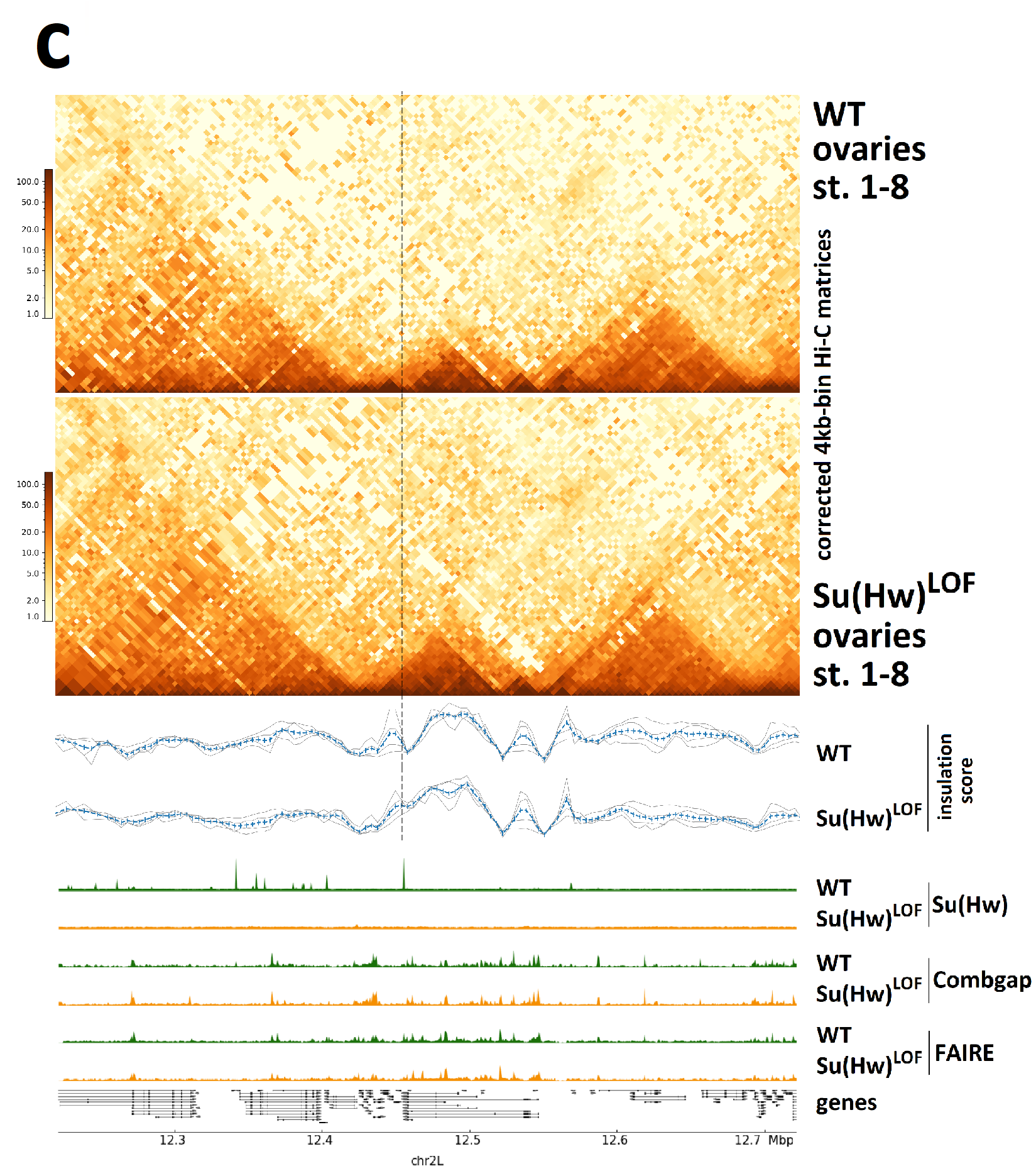


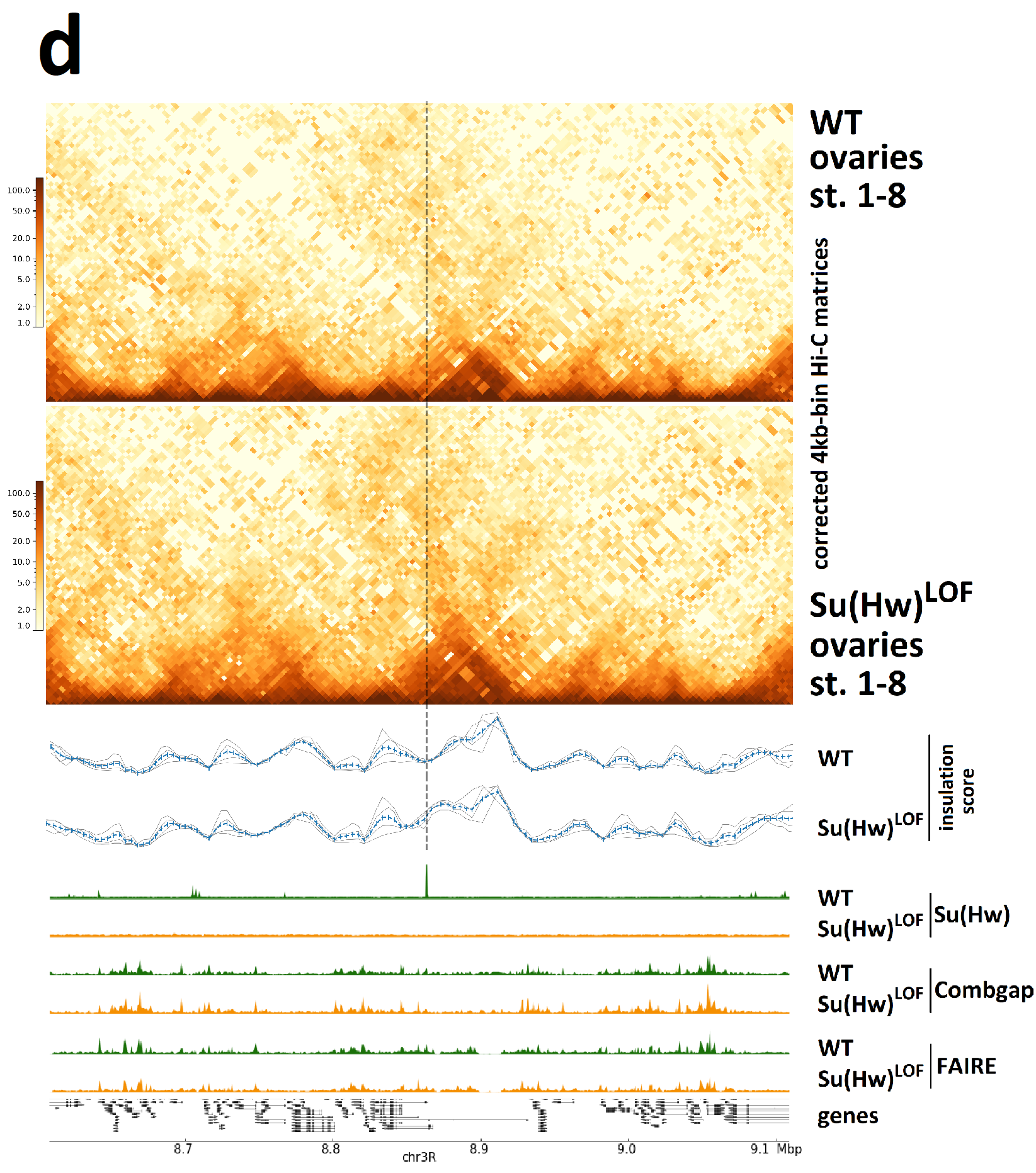


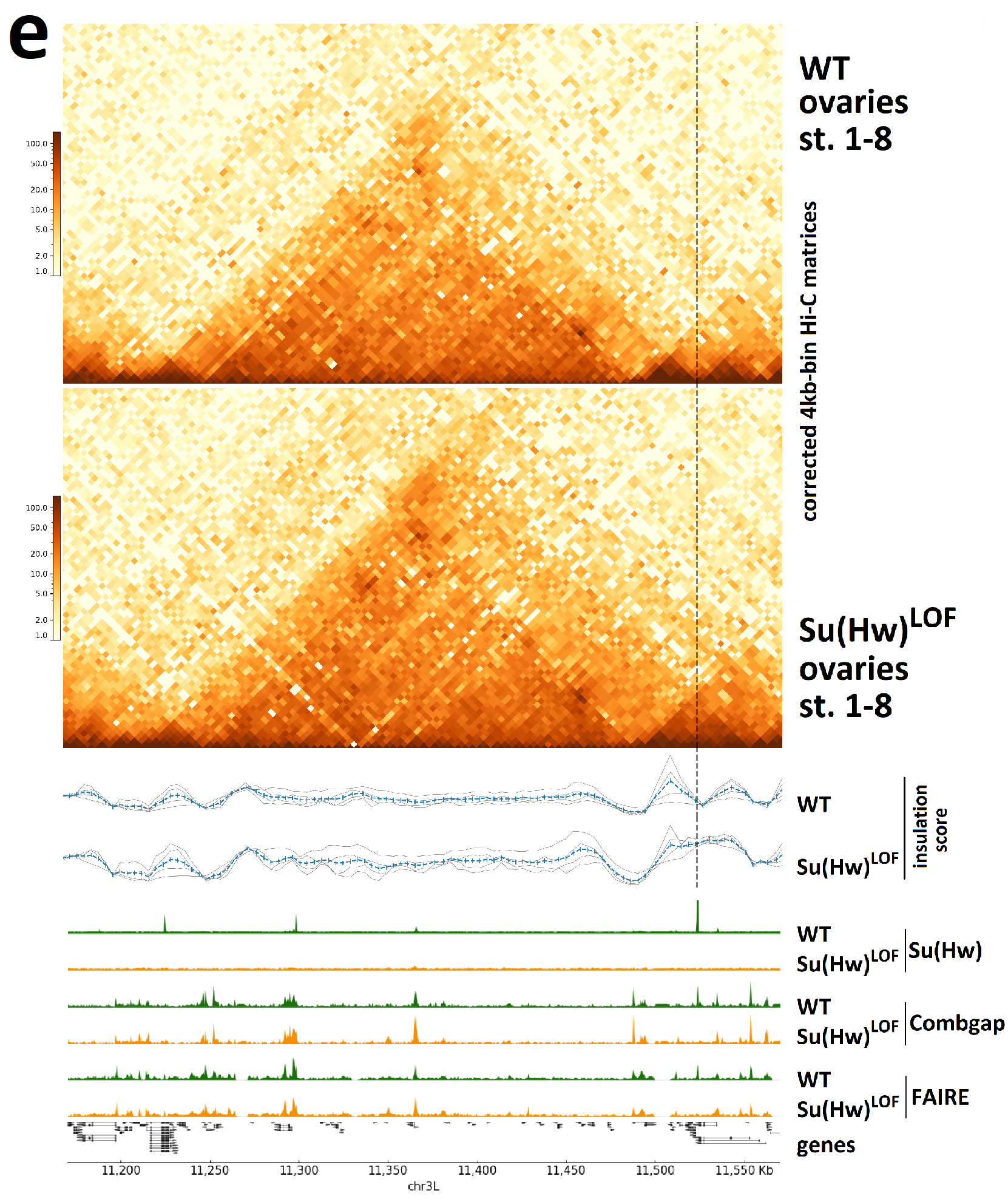


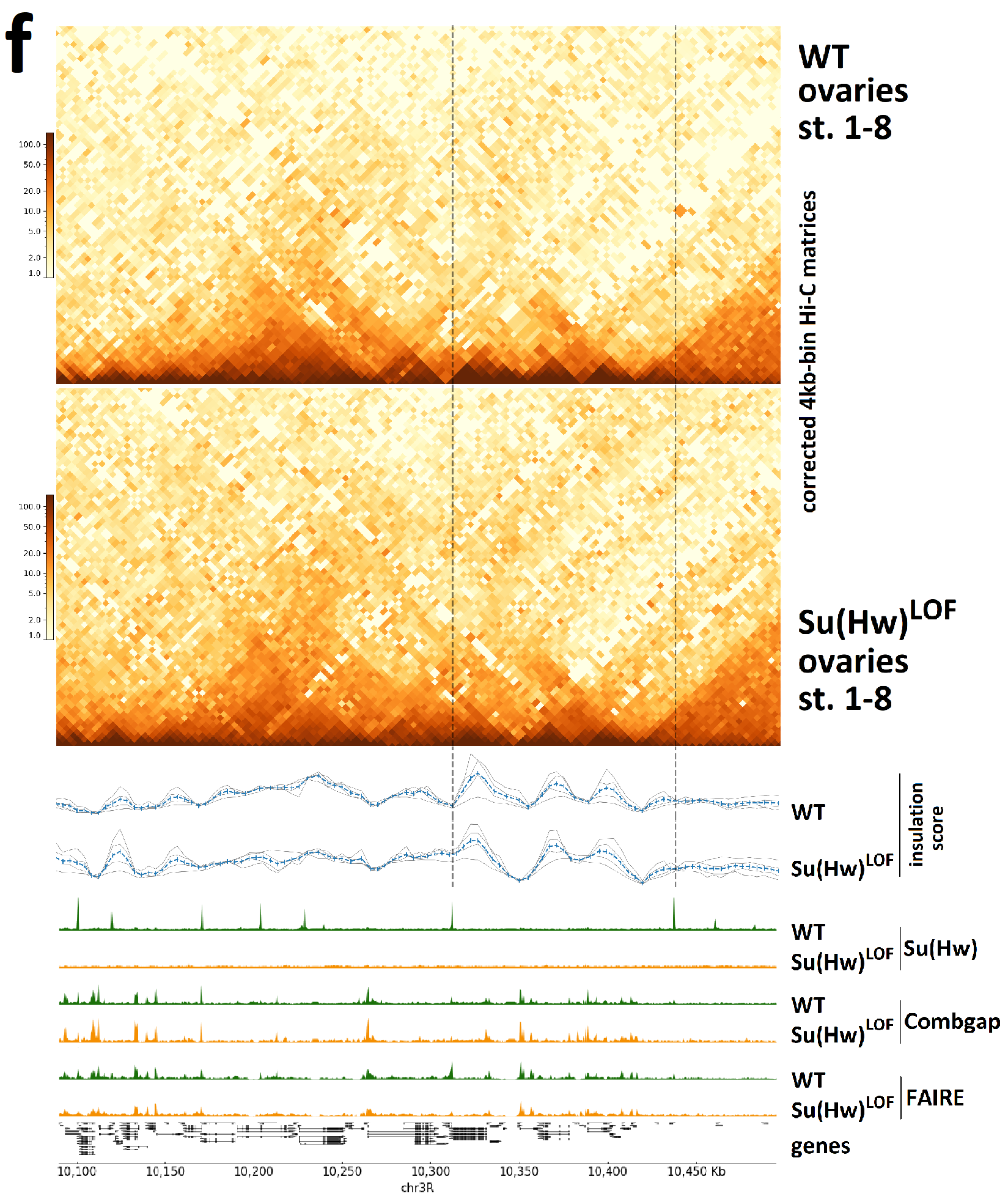


**
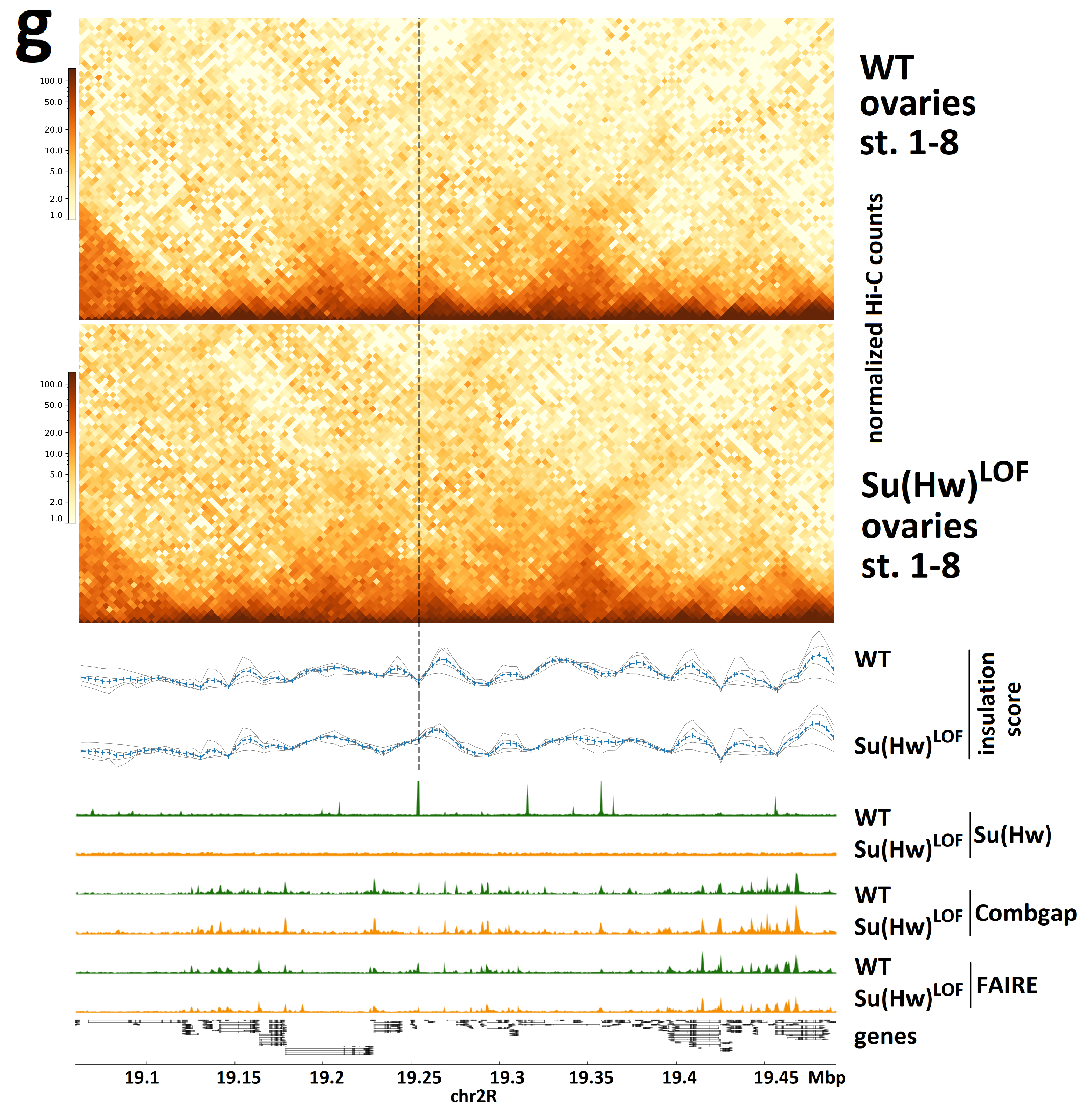
**

**Supplementary figure 5. LRIs between Su(Hw) and Combgap ChIP-Seq peaks.**

**(a)** Averaged spatial interactions in the sets of direct Su(Hw), indirect Su(Hw) and Rpb3 (positive control) ChIP-peaks in the wild-type (WT) and Su(Hw)^LOF^ ovaries, estimated with a coolpup.py program. The minimal and maximal distances of interactions were set at 200 kbp and 1000 kbp, correspondingly, the pad size is ±40 kb around the central pixel.

**(b)** Averaged spatial interactions between the different sets of Su(Hw), BEAF32 and M1BP ChIP-Seq peaks in the wild-type (WT) and Su(Hw)^LOF^ ovaries, estimated with a coolpup.py program. The minimal and maximal distances of interactions were set at 200 kbp and 1000 kbp, correspondingly, the pad size is ±40 kb around the central pixel.

**(c-g)** Hi-C matrices from the wild-type (WT) and Su(Hw)^LOF^ ovaries (on top), showing two genomic region with insulator scores and occupancies of Su(Hw), Combgap (ChIP-Seq) and open chromatin regions (according to FAIRE-Seq) in the wild-type (WT) and Su(Hw)^LOF^ ovaries. The image was generated using pyGenomeTracks. This particular regions were selected to illustrate Su(Hw)-dependence of long range interactions (LRIs) between direct Su(Hw) FAIRE+ and Combgap ChIP-Seq peaks (position of direct Su(Hw) FAIRE+ peaks in these LRIs are marked with the vertical lines).


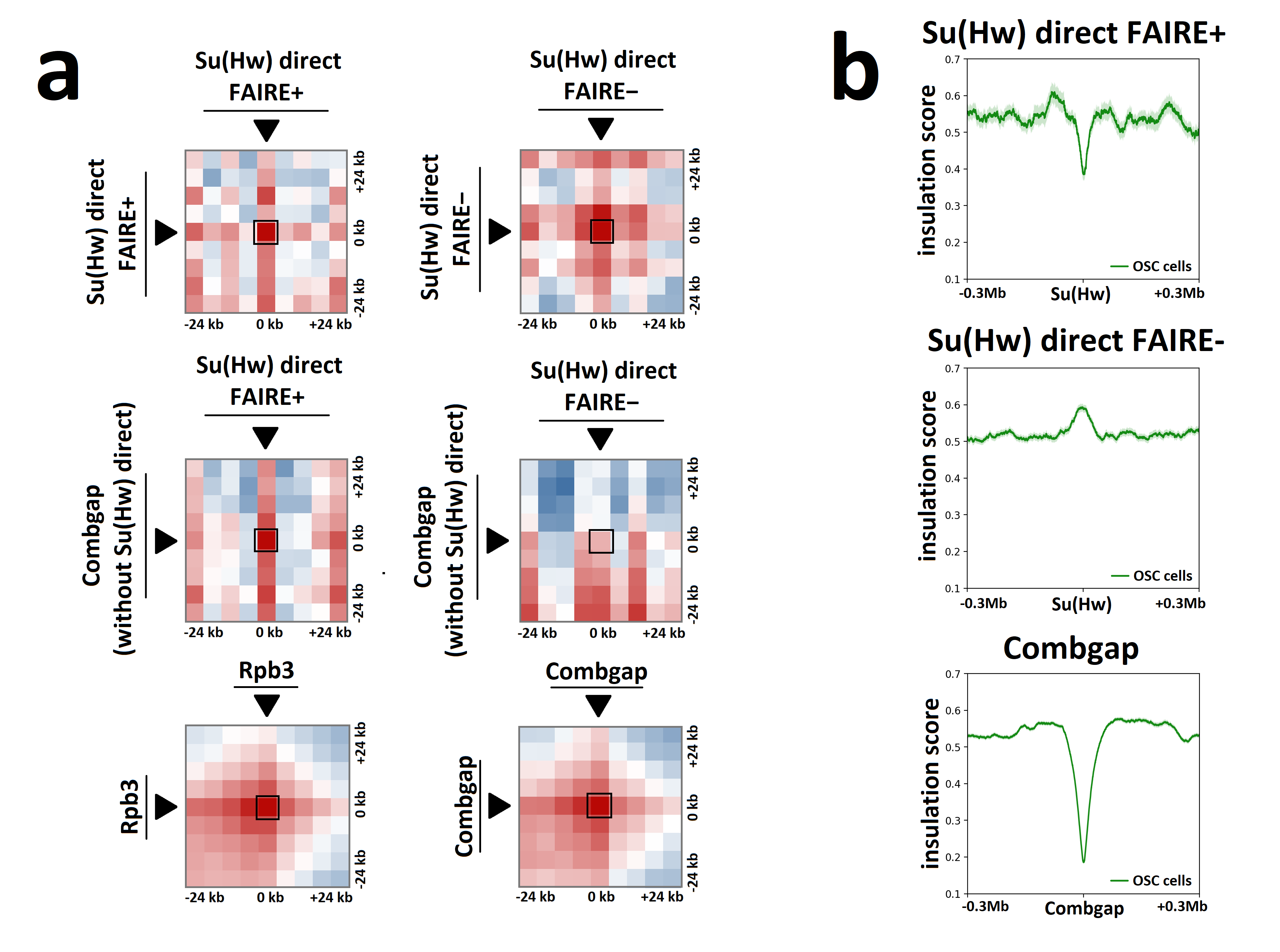


**Supplementary figure 6. LRIs between Su(Hw) and Combgap ChIP-Seq peaks from *Drosophila* ovarian somatic cell (OSC) line Hi-C.**

**(a)** Averaged spatial interactions in the sets of direct Su(Hw) FAIRE+, direct Su(Hw) FAIRE-, Combgap and Rpb3 (positive control) ChIP-peaks from the wild-type ovaries estimated in Hi-C from EGFP dsRNA treated control OSC cells (from Iwasaki et al (2021) EMBO J, 40(18):e108345) with a coolpup.py program. The minimal and maximal distances of interactions were set at 200 kbp and 1000 kbp, correspondingly, the pad size is ±24 kb around the central pixel.

**(b)** Profile plots of insulation score at direct Su(Hw) FAIRE+, Su(Hw) FAIRE- and Combgap ChIP-Seq peaks from the wild-type ovaries, calculated using Hi-C from EGFP dsRNA treated control OSC cells (from Iwasaki et al (2021) EMBO J, 40(18):e108345). The pile-up profiles were generated as a median of insulation score signal. The standard error is displayed on the profiles as semi-transparent area around the main line of the profiles.


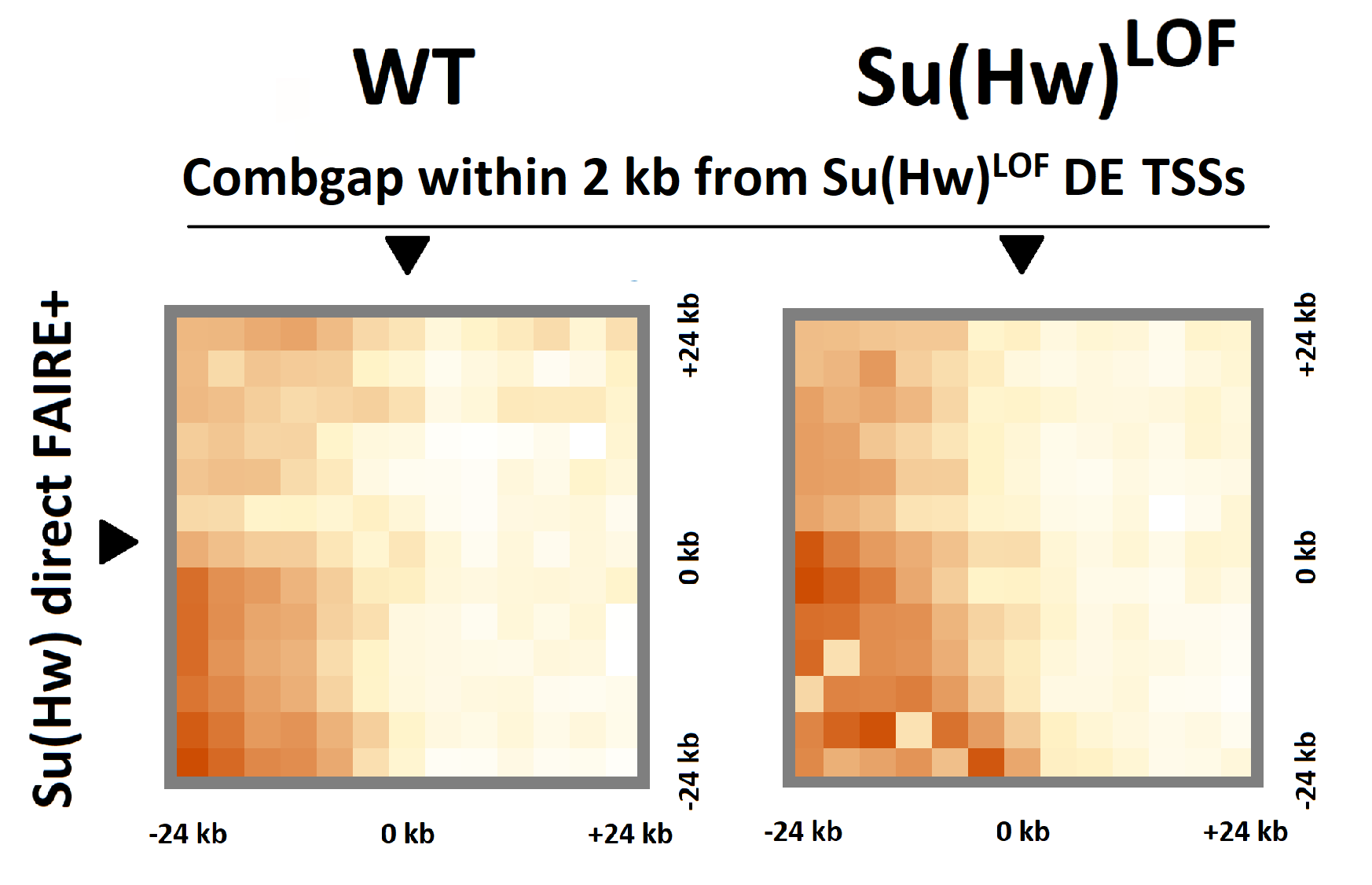


**Supplementary figure 7. Averaged spatial interactions between the sets of direct Su(Hw) FAIRE+ ChIP-Seq peaks and Combgap ChIP-Seq peaks located within 2kb from TSSs of Su(Hw)^LOF^ DE genes in the wild-type (WT) and Su(Hw)^LOF^ ovaries, estimated with a coolpup.py program.** The minimal and maximal distances of interactions were set at 50 kb and 200 kb, correspondingly, the pad size is ±24 kb around the central pixel.


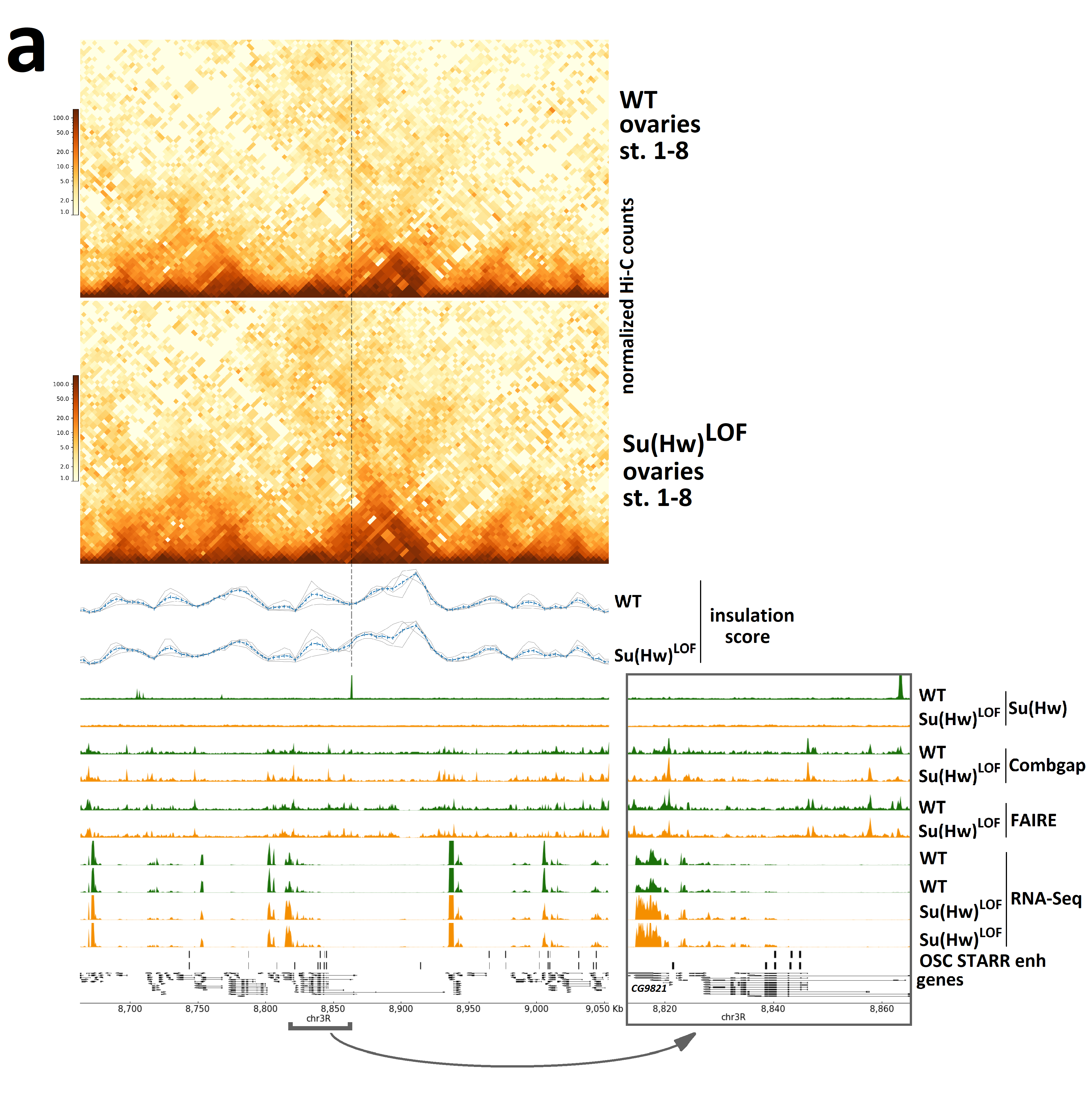


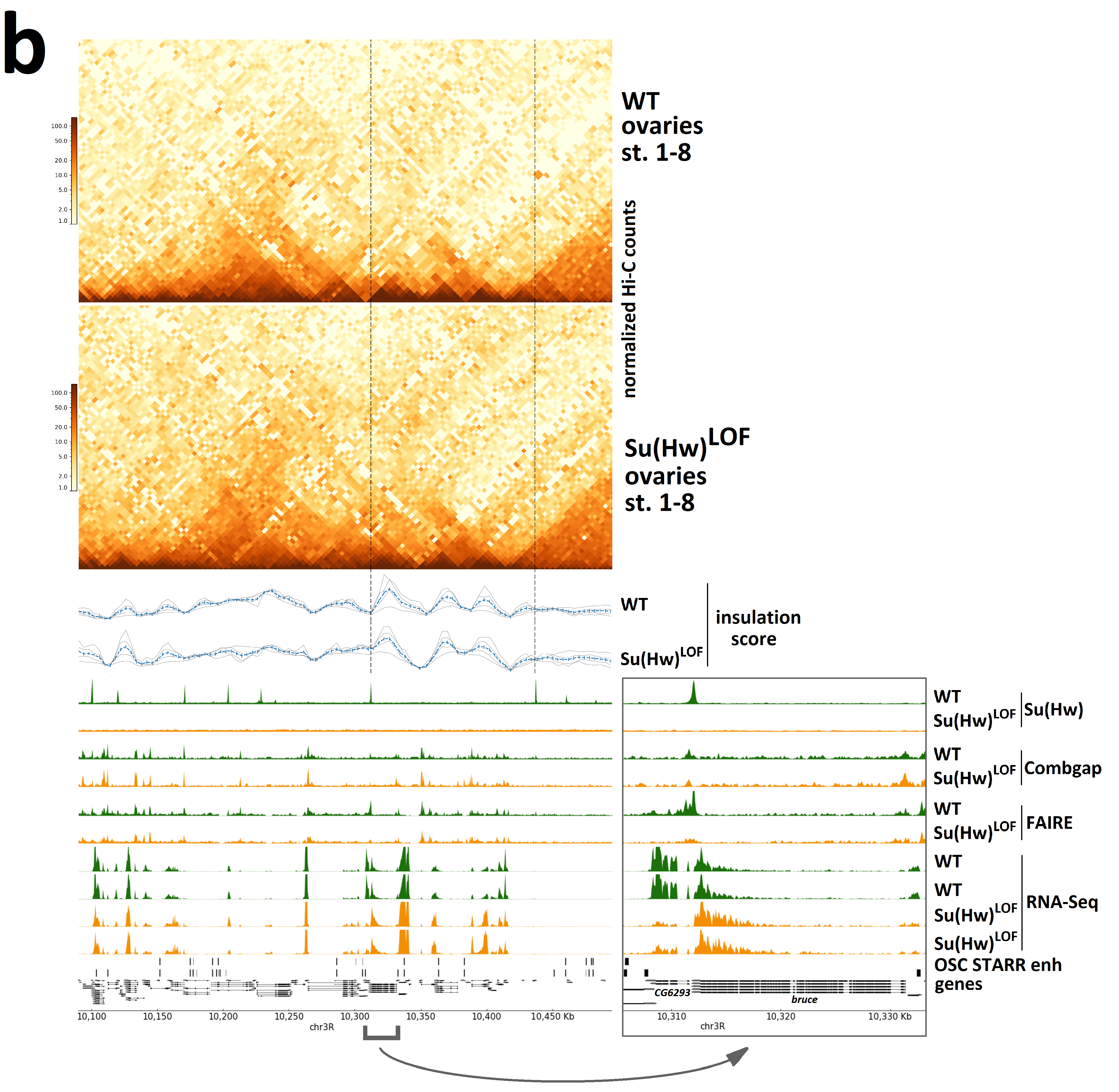


**Supplementary figure 8. LRIs between Su(Hw) and Combgap ChIP-Seq peaks correlate with transcription mis-regulation in Su(Hw)^LOF^ background. (a-b)** Hi-C matrices from the wild-type (WT) and Su(Hw)^LOF^ ovaries (on top), showing four genomic region with insulator scores and occupancies of Su(Hw), Combgap (ChIP-seq), open chromatin regions (FAIRE-Seq), and gene expression (RNA-Seq) in the wild-type (WT) and Su(Hw)^LOF^ ovaries. The image was generated using pyGenomeTracks. For RNA-Seq two biological replicates are shown for each genotype. This particular regions were selected to illustrate correlation between the changes in long range interactions between direct Su(Hw) FAIRE+ and Combgap ChIP-Seq peaks and gene expression (positions of direct Su(Hw) FAIRE+ peaks in these LRIs are marked with dashed grey lines). The Su(Hw)^LOF^ mis-regulated genes are indicated on the areas enlarged near direct Su(Hw) FAIRE+ peaks (on the right from each figure).


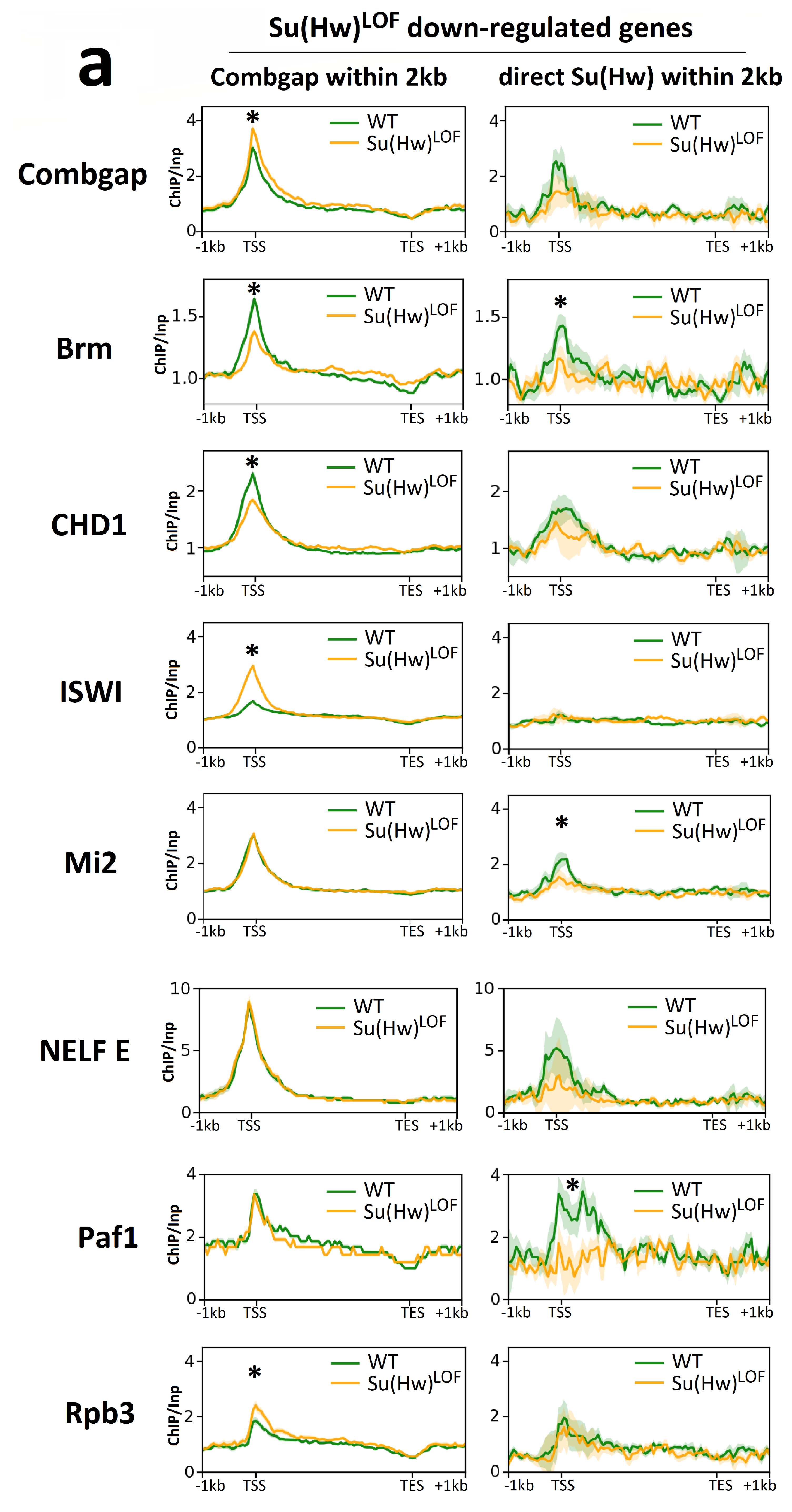


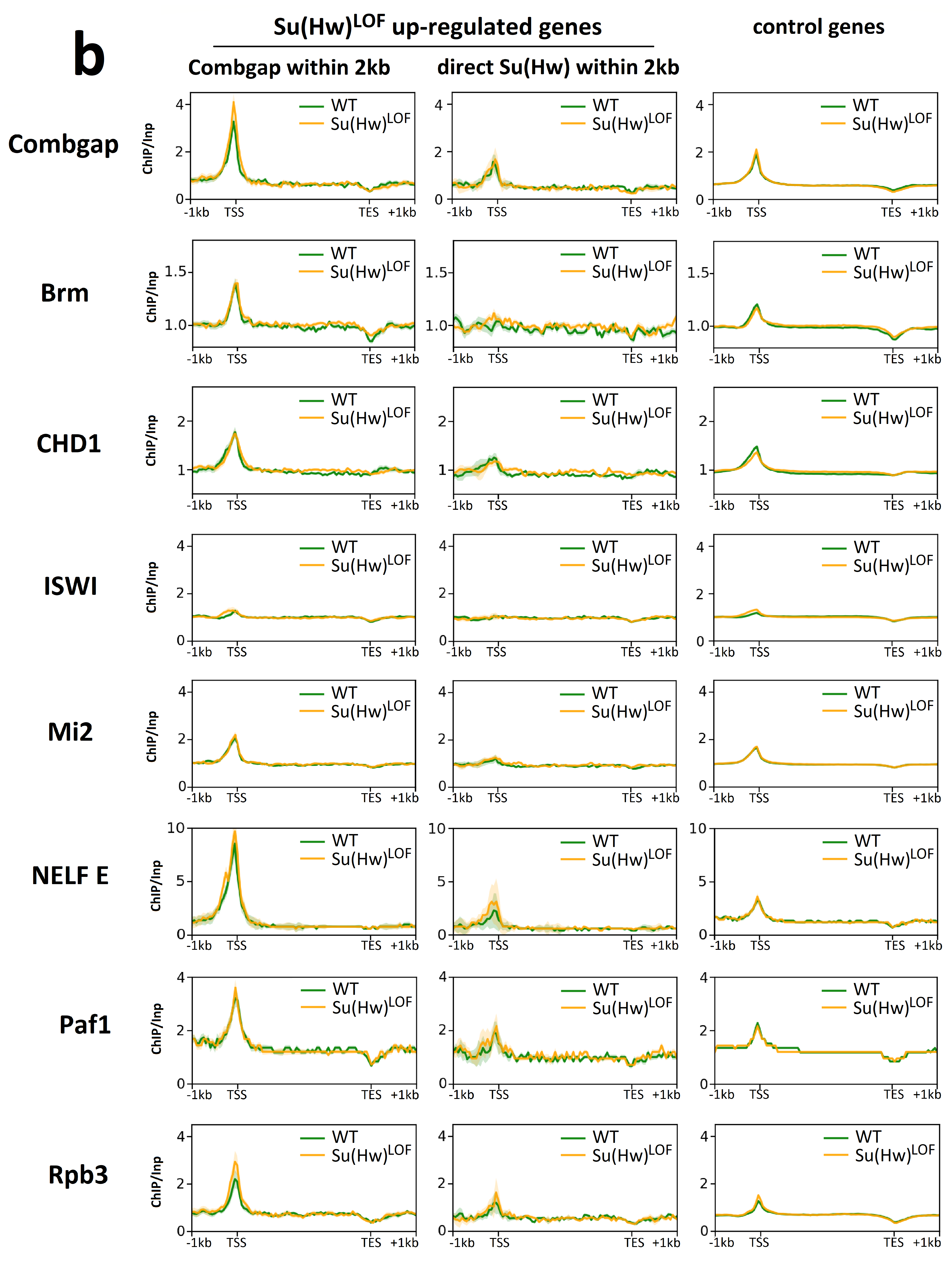


**Supplementary figure 9. Average profiles of Combgap, Brm, CHD1, ISWI, Mi-2, NELF E, Paf1 and Rpb3 ChIP/Input signals on the genes, down-regulated (a) and up-regulated (b) in Su(Hw)^LOF^ ovaries compared to the wild-type.** The pile-up profiles were generated as a median of ChIP/Input signal. The significant changes in ChIP/Input signal upon Su(Hw)^LOF^ are marked with *. As a set of control we used genes, expression of which does not change significantly in Su(Hw)^LOF^ ovaries compared to the wild-type (fold-change < 2). The standard error is displayed on the profiles as semi-transparent area around the main line of the profiles.


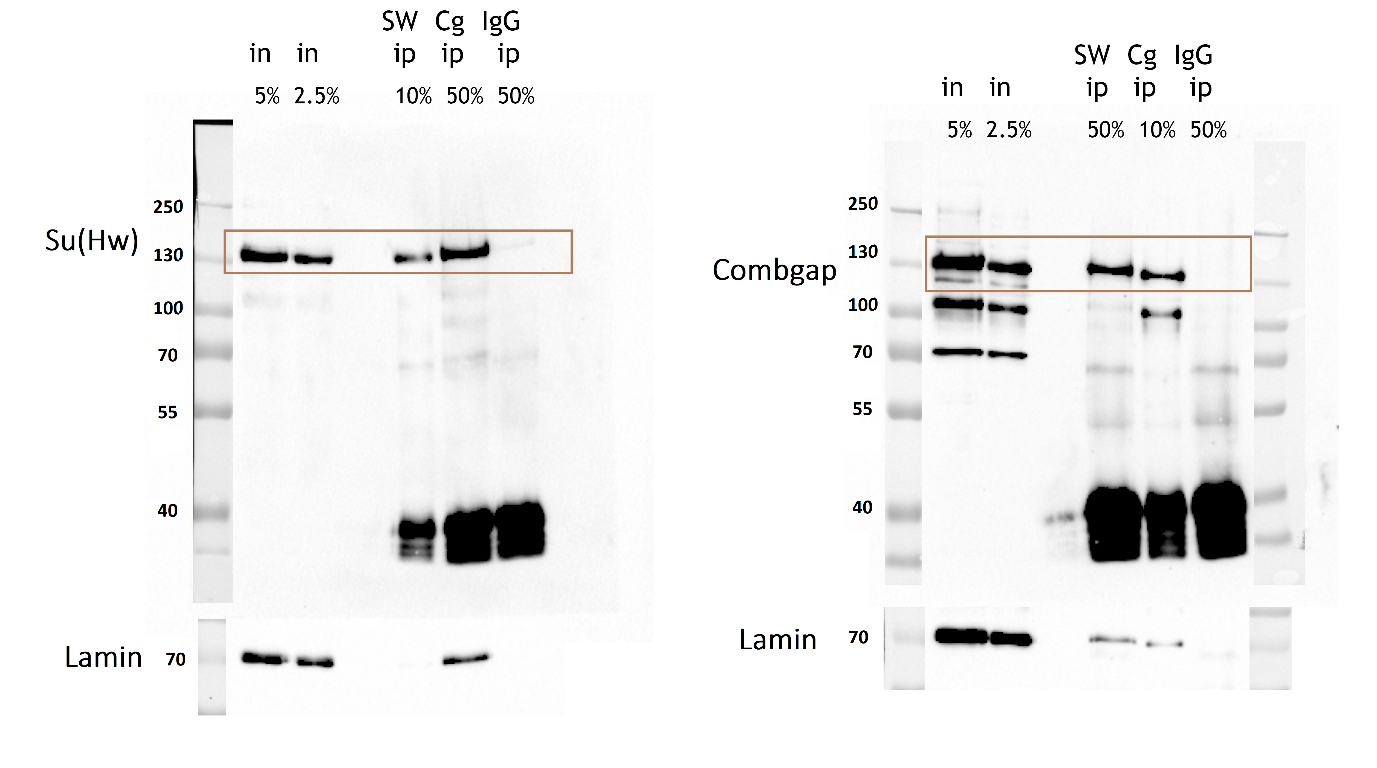


**Supplementary figure 10. The full Western blots used in Fig. 1d.** The IPs were performed with antibodies against Combgap and Su(Hw) (a serum of non-immunized rabbits (ip IgG) was used as a negative control), which is indicated on the top of the figure. Western blots were stained with the corresponding antibodies indicated on the left of the figures. Anti-lamin staining was used as loading control. All input and IP samples were loaded on a single western blot. The numbers above the inputs represent a portion of a loaded fraction (in respect to the amount used for immunoprecipitations). The brown frames indicate the regions of the blots with specific antibody staining that were presented at Fig. 1d.

**Supplementary Table 1. Transcripts that are significantly differentially expressed in Su(Hw)^LOF^ ovaries (with adjusted p-value < 0.01 and fold-change (FC) > 2)**

**(a) The list of Su(Hw)^LOF^ up-regulated transcripts**

| ENSEMBLTRANS | SYMBOL | logFC | Average Expression | Adjusted  P-value |
| --- | --- | --- | --- | --- |
| FBtr0081088 | ncm | -1,3888 | 10,2231 | 0,0035 |
| FBtr0310323 | CG13185 | -1,1602 | 9,7655 | 0,0037 |
| FBtr0076457 | aay | -1,6668 | 9,0552 | 0,0035 |
| FBtr0329922 | qin | -1,1528 | 7,9212 | 0,0077 |
| FBtr0290134 | CG42232 | -1,1195 | 7,8927 | 0,0038 |
| FBtr0083154 | AOX1 | -1,0067 | 7,7839 | 0,0045 |
| FBtr0088226 | RpS15Ab | -1,0195 | 7,7063 | 0,0075 |
| FBtr0079466 | CG5958 | -3,3924 | 7,5849 | 0,0044 |
| FBtr0302586 | RpL3 | -1,3484 | 7,5559 | 0,0076 |
| FBtr0301163 | Pih1D1 | -1,2592 | 7,5032 | 0,0077 |
| FBtr0073414 | Imp | -1,3201 | 7,1553 | 0,0073 |
| FBtr0071498 | Atg8a | -2,3524 | 6,9677 | 0,0037 |
| FBtr0086897 | rhi | -1,4345 | 6,9540 | 0,0099 |
| FBtr0302084 | lbk | -1,1683 | 6,9344 | 0,0038 |
| FBtr0082287 | CG4570 | -1,9567 | 6,9098 | 0,0038 |
| FBtr0310385 | Su(var)3-9 | -1,0788 | 6,5661 | 0,0078 |
| FBtr0273437 | Cubn | -2,0762 | 6,5571 | 0,0061 |
| FBtr0073270 | Chd64 | -1,2763 | 6,5489 | 0,0089 |
| FBtr0074635 | Atg101 | -1,0654 | 6,5053 | 0,0069 |
| FBtr0084289 | CG5346 | -1,7111 | 6,4107 | 0,0037 |
| FBtr0072064 | Pi3K59F | -1,1357 | 6,2586 | 0,0076 |
| FBtr0335275 | Src42A | -1,1175 | 6,2241 | 0,0064 |
| FBtr0302027 | Mrp4 | -1,9097 | 6,2158 | 0,0053 |
| FBtr0074684 | CG8034 | -2,4991 | 6,1776 | 0,0051 |
| FBtr0300082 | wech | -1,9308 | 6,1684 | 0,0054 |
| FBtr0085715 | CG15547 | -3,8401 | 6,1468 | 0,0058 |
| FBtr0078267 | fng | -1,6833 | 6,0980 | 0,0038 |
| FBtr0332210 | kek1 | -1,8697 | 6,0166 | 0,0035 |
| FBtr0071661 | CG10082 | -1,4458 | 5,9340 | 0,0077 |
| FBtr0070406 | Ilp6 | -3,6480 | 5,8837 | 0,0035 |
| FBtr0074884 | kto | -1,2508 | 5,8512 | 0,0062 |
| FBtr0299657 | bun | -3,1342 | 5,8231 | 0,0071 |
| FBtr0085110 | Men-b | -1,0716 | 5,8004 | 0,0053 |
| FBtr0075657 | sstn | -1,5564 | 5,7214 | 0,0084 |
| FBtr0078274 | Six4 | -1,6973 | 5,6531 | 0,0071 |
| FBtr0082233 | CG12592 | -3,9369 | 5,5693 | 0,0045 |
| FBtr0076106 | CG32091 | -2,7929 | 5,5461 | 0,0038 |
| FBtr0082747 | Dic1 | -1,6715 | 5,5411 | 0,0043 |
| FBtr0082569 | GstD2 | -4,7833 | 5,3906 | 0,0038 |
| FBtr0331689 | CG17801 | -1,0767 | 5,3758 | 0,0061 |
| FBtr0339612 | CG12290 | -2,2651 | 5,2835 | 0,0088 |
| FBtr0084702 | sosie | -4,6768 | 5,2513 | 0,0054 |
| FBtr0301313 | Zasp52 | -2,5944 | 4,9666 | 0,0052 |
| FBtr0073069 | CG14961 | -2,4982 | 4,9268 | 0,0077 |
| FBtr0085631 | Fer1HCH | -1,1851 | 4,9006 | 0,0077 |
| FBtr0344717 | Akt1 | -1,3427 | 4,8265 | 0,0091 |
| FBtr0085803 | CycG | -1,3274 | 4,7509 | 0,0076 |
| FBtr0070681 | pon | -1,7891 | 4,6657 | 0,0077 |
| FBtr0070183 | CG11638 | -2,1028 | 4,6242 | 0,0044 |
| FBtr0077156 | Uev1A | -1,0919 | 4,4882 | 0,0085 |
| FBtr0303999 | Tao | -1,1919 | 4,4537 | 0,0077 |
| FBtr0114586 | egl | -1,4769 | 4,4338 | 0,0090 |
| FBtr0072561 | Reg-2 | -1,6310 | 4,4241 | 0,0054 |
| FBtr0087893 | CG17739 | -4,5770 | 4,3682 | 0,0074 |
| FBtr0076136 | CG6071 | -2,6782 | 4,3439 | 0,0042 |
| FBtr0075811 | Tgi | -2,2931 | 4,1782 | 0,0044 |
| FBtr0088936 | Gdap2 | -2,3574 | 4,1681 | 0,0044 |
| FBtr0302711 | CG42673 | -1,7837 | 4,1540 | 0,0065 |
| FBtr0303781 | CG17732 | -1,4477 | 4,1479 | 0,0079 |
| FBtr0075023 | CG6836 | -2,5009 | 4,1262 | 0,0042 |
| FBtr0076808 | Mcad | -1,5739 | 4,0957 | 0,0068 |
| FBtr0082277 | Rbp1 | -1,1741 | 4,0770 | 0,0096 |
| FBtr0072742 | Pcyt2 | -2,4185 | 4,0732 | 0,0047 |
| FBtr0076724 | ERR | -1,9200 | 4,0589 | 0,0052 |
| FBtr0078216 | CG5059 | -3,2331 | 3,9578 | 0,0038 |
| FBtr0331790 | fmt | -1,6375 | 3,8935 | 0,0063 |
| FBtr0078506 | CG11426 | -2,6043 | 3,8501 | 0,0065 |
| FBtr0075424 | Cpr72Ec | -2,4147 | 3,8341 | 0,0065 |
| FBtr0310343 | CG42450 | -2,5921 | 3,7984 | 0,0080 |
| FBtr0304867 | CG43066 | -1,7291 | 3,7728 | 0,0091 |
| FBtr0073728 | CG32640 | -1,5343 | 3,7099 | 0,0076 |
| FBtr0290036 | stas | -2,3767 | 3,6920 | 0,0077 |
| FBtr0340007 | CG30089 | -1,2312 | 3,6772 | 0,0091 |
| FBtr0333347 | Nhe3 | -2,0745 | 3,6668 | 0,0092 |
| FBtr0084536 | Pisd | -2,1152 | 3,6535 | 0,0045 |
| FBtr0088107 | CG13227 | -2,5734 | 3,6115 | 0,0044 |
| FBtr0332936 | CG1695 | -8,8209 | 3,5730 | 0,0093 |
| FBtr0073729 | CG32640 | -2,4192 | 3,5546 | 0,0042 |
| FBtr0332386 | CG42340 | -2,4654 | 3,5391 | 0,0044 |
| FBtr0080287 | Ada1-2 | -2,4736 | 3,5272 | 0,0074 |
| FBtr0089855 | rho-5 | -1,9616 | 3,5186 | 0,0052 |
| FBtr0072239 | CG4797 | -3,7264 | 3,4488 | 0,0044 |
| FBtr0086180 | Tsp42Ee | -1,5648 | 3,4063 | 0,0072 |
| FBtr0071036 | ogre | -1,6172 | 3,3953 | 0,0071 |
| FBtr0304994 | Sarm | -1,6984 | 3,3153 | 0,0063 |
| FBtr0083791 | CG11453 | -5,4819 | 3,3043 | 0,0091 |
| FBtr0088525 | RpL31 | -2,2110 | 3,2921 | 0,0091 |
| FBtr0304622 | ths | -2,1171 | 3,2547 | 0,0062 |
| FBtr0334498 | orb | -2,5699 | 3,1501 | 0,0052 |
| FBtr0303874 | Apoltp | -1,6989 | 3,0749 | 0,0086 |
| FBtr0343610 | Npc1a | -1,7278 | 3,0496 | 0,0098 |
| FBtr0333726 | Fas1 | -2,4754 | 3,0319 | 0,0052 |
| FBtr0085180 | TTLL6B | -1,8717 | 3,0278 | 0,0079 |
| FBtr0304054 | Esp | -2,9045 | 2,9507 | 0,0051 |
| FBtr0087370 | scb | -2,5239 | 2,8231 | 0,0082 |
| FBtr0085150 | CG6277 | -2,1461 | 2,7703 | 0,0069 |
| FBtr0333664 | msi | -1,8757 | 2,7564 | 0,0078 |
| FBtr0075983 | SCaMC | -2,6667 | 2,7111 | 0,0048 |
| FBtr0073916 | CG9411 | -1,5735 | 2,6895 | 0,0088 |
| FBtr0076164 | nkt | -3,0604 | 2,6081 | 0,0063 |
| FBtr0076885 | RhoGEF4 | -2,1226 | 2,4870 | 0,0061 |
| FBtr0076126 | CG11658 | -2,6459 | 2,4559 | 0,0049 |
| FBtr0290076 | kay | -2,9489 | 2,4218 | 0,0052 |
| FBtr0071653 | Egfr | -1,8659 | 2,3943 | 0,0073 |
| FBtr0308682 | CG13004 | -3,0548 | 2,3697 | 0,0091 |
| FBtr0303757 | Kank | -2,2837 | 2,3634 | 0,0063 |
| FBtr0080527 | CG18507 | -2,0327 | 2,2772 | 0,0090 |
| FBtr0112812 | dlg1 | -2,2395 | 2,1770 | 0,0083 |
| FBtr0331535 | CG10089 | -3,1348 | 2,1287 | 0,0086 |
| FBtr0335385 | CG17261 | -2,2891 | 2,1213 | 0,0071 |
| FBtr0074048 | Alp11 | -3,1431 | 2,0637 | 0,0045 |
| FBtr0076485 | CG3982 | -3,0797 | 2,0311 | 0,0061 |
| FBtr0331700 | CG7362 | -3,4476 | 2,0255 | 0,0051 |
| FBtr0089426 | CG18853 | -6,8745 | 1,9872 | 0,0061 |
| FBtr0100469 | CG3662 | -2,8416 | 1,9800 | 0,0076 |
| FBtr0332661 | Oseg2 | -3,1179 | 1,9797 | 0,0063 |
| FBtr0072402 | emp | -2,8533 | 1,9179 | 0,0084 |
| FBtr0072255 | AANAT1 | -3,0869 | 1,8441 | 0,0061 |
| FBtr0081771 | CG2993 | -4,0338 | 1,8323 | 0,0051 |
| FBtr0070340 | Sik2 | -2,2460 | 1,8196 | 0,0073 |
| FBtr0079127 | tomb | -2,8946 | 1,8002 | 0,0077 |
| FBtr0301417 | IA-2 | -2,6028 | 1,7298 | 0,0061 |
| FBtr0303106 | Dhc62B | -2,9648 | 1,7290 | 0,0076 |
| FBtr0081118 | CG33120 | -2,9123 | 1,7227 | 0,0052 |
| FBtr0083790 | CG11407 | -3,9742 | 1,7065 | 0,0095 |
| FBtr0112846 | Hnf4 | -4,1836 | 1,5770 | 0,0050 |
| FBtr0310433 | Tpst | -3,6220 | 1,5764 | 0,0044 |
| FBtr0340635 | chas | -3,8381 | 1,5564 | 0,0044 |
| FBtr0077478 | ft | -2,5766 | 1,5545 | 0,0073 |
| FBtr0089418 | Cyp12d1-d | -7,8101 | 1,4967 | 0,0078 |
| FBtr0307203 | CG9593 | -2,3972 | 1,4549 | 0,0077 |
| FBtr0075282 | rogdi | -3,6065 | 1,4127 | 0,0096 |
| FBtr0077686 | CG3104 | -5,2666 | 1,3756 | 0,0042 |
| FBtr0113135 | ens | -3,1602 | 1,2257 | 0,0069 |
| FBtr0085496 | CG31038 | -2,8838 | 1,0769 | 0,0066 |
| FBtr0072563 | Kah | -2,2832 | 1,0211 | 0,0093 |
| FBtr0307508 | CG31817 | -3,3111 | 1,0156 | 0,0099 |
| FBtr0333219 | olf413 | -3,2914 | 0,9561 | 0,0092 |
| FBtr0344333 | Hph | -2,6591 | 0,9509 | 0,0077 |
| FBtr0113389 | CG31103 | -4,4141 | 0,9437 | 0,0089 |
| FBtr0302283 | Trpml | -7,9001 | 0,9260 | 0,0065 |
| FBtr0075931 | CG32110 | -3,1265 | 0,9032 | 0,0089 |
| FBtr0075435 | Notum | -3,6786 | 0,8350 | 0,0072 |
| FBtr0083810 | CG6231 | -2,6756 | 0,8282 | 0,0092 |
| FBtr0080811 | Tep1 | -6,0747 | 0,8002 | 0,0038 |
| FBtr0299573 | CG42260 | -5,2976 | 0,7903 | 0,0044 |
| FBtr0303998 | Ilp6 | -4,3543 | 0,6774 | 0,0044 |
| FBtr0078591 | Osi24 | -3,3059 | 0,6318 | 0,0077 |
| FBtr0074952 | Trpml | -8,7238 | 0,5334 | 0,0042 |
| FBtr0082084 | CG18542 | -7,1050 | 0,5201 | 0,0045 |
| FBtr0340086 | Pkcdelta | -3,0049 | 0,4959 | 0,0085 |
| FBtr0077394 | hoe2 | -4,1603 | 0,4671 | 0,0068 |
| FBtr0087651 | CG6209 | -2,5810 | 0,4616 | 0,0091 |
| FBtr0081883 | CG8036 | -3,3742 | 0,4342 | 0,0062 |
| FBtr0080623 | ms(2)34Fe | -2,8904 | 0,4302 | 0,0077 |
| FBtr0089963 | Tm1 | -2,8904 | 0,4302 | 0,0077 |
| FBtr0273192 | CG3339 | -3,7774 | 0,3803 | 0,0079 |
| FBtr0086171 | Tsp42Ea | -8,3107 | 0,3539 | 0,0061 |
| FBtr0333154 | hwt | -6,4516 | 0,1853 | 0,0058 |
| FBtr0344825 | Gem4b | -4,0149 | 0,1425 | 0,0059 |
| FBtr0082085 | CG18542 | -7,8617 | 0,1131 | 0,0035 |
| FBtr0084845 | Muc96D | -3,8779 | 0,0729 | 0,0063 |
| FBtr0075188 | CG32182 | -3,8829 | 0,0680 | 0,0096 |
| FBtr0329896 | mthl8 | -7,6914 | 0,0361 | 0,0038 |
| FBtr0113333 | CG12355 | -2,8089 | -0,0950 | 0,0098 |
| FBtr0334906 | betaTub97EF | -3,3825 | -0,1629 | 0,0100 |
| FBtr0070599 | Crg-1 | -7,2720 | -0,1718 | 0,0044 |
| FBtr0086929 | CG30103 | -5,5478 | -0,2683 | 0,0076 |
| FBtr0333065 | jeb | -3,1691 | -0,2793 | 0,0092 |
| FBtr0080871 | CG4631 | -3,6033 | -0,4307 | 0,0064 |
| FBtr0085453 | Gycalpha99B | -5,0696 | -0,4944 | 0,0080 |
| FBtr0081549 | alpha-Est6 | -3,4034 | -0,5397 | 0,0091 |
| FBtr0273260 | CG11898 | -3,4034 | -0,5397 | 0,0091 |
| FBtr0302291 | PPO1 | -4,9220 | -0,5718 | 0,0081 |
| FBtr0076982 | GluRIA | -6,4805 | -0,5944 | 0,0062 |
| FBtr0331441 | CG3609 | -5,8565 | -0,8980 | 0,0042 |
| FBtr0307033 | Nlg2 | -5,6168 | -1,0051 | 0,0038 |
| FBtr0080252 | kek2 | -5,5028 | -1,0885 | 0,0094 |
| FBtr0344276 | Unc-115a | -5,4711 | -1,0975 | 0,0062 |
| FBtr0076718 | CG7213 | -5,0956 | -1,2895 | 0,0086 |
| FBtr0342919 | CG17321 | -5,0066 | -1,3068 | 0,0046 |
| FBtr0083864 | CG10887 | -4,9633 | -1,3179 | 0,0087 |
| FBtr0082376 | Ugt35E2 | -4,9571 | -1,3533 | 0,0059 |
| FBtr0302199 | GlcAT-S | -4,9320 | -1,3580 | 0,0044 |
| FBtr0083671 | VAChT | -4,8754 | -1,3724 | 0,0049 |
| FBtr0087485 | Dh44-R1 | -4,8803 | -1,3757 | 0,0044 |
| FBtr0333355 | nrv3 | -4,9050 | -1,3773 | 0,0053 |
| FBtr0339951 | CG6329 | -4,8491 | -1,4031 | 0,0051 |
| FBtr0086238 | dpr1 | -4,8623 | -1,4049 | 0,0086 |
| FBtr0087756 | CG4714 | -4,8093 | -1,4085 | 0,0045 |
| FBtr0088503 | Or45a | -4,8067 | -1,4158 | 0,0044 |
| FBtr0082820 | ninaB | -4,8102 | -1,4290 | 0,0072 |
| FBtr0082745 | PK2-R1 | -4,7311 | -1,4446 | 0,0051 |
| FBtr0080786 | CG18109 | -4,7357 | -1,4486 | 0,0044 |
| FBtr0084949 | boss | -4,7105 | -1,4486 | 0,0063 |
| FBtr0307079 | CG18641 | -4,6442 | -1,4847 | 0,0059 |
| FBtr0307538 | RSG7 | -4,6126 | -1,4938 | 0,0095 |
| FBtr0289998 | side | -4,6293 | -1,5126 | 0,0052 |
| FBtr0072029 | CG3530 | -4,6293 | -1,5126 | 0,0052 |
| FBtr0086165 | CG30158 | -4,6441 | -1,5150 | 0,0099 |
| FBtr0301558 | Rph | -4,5706 | -1,5248 | 0,0052 |
| FBtr0332932 | CG43901 | -4,5706 | -1,5248 | 0,0052 |
| FBtr0085347 | DIP-gamma | -4,5582 | -1,5454 | 0,0051 |
| FBtr0081327 | sNPF | -4,5582 | -1,5454 | 0,0051 |
| FBtr0300354 | CG15635 | -4,5582 | -1,5454 | 0,0051 |
| FBtr0080675 | CG3491 | -4,5582 | -1,5454 | 0,0051 |
| FBtr0088495 | CG1688 | -4,4800 | -1,5815 | 0,0051 |
| FBtr0303203 | CG42741 | -4,4800 | -1,5815 | 0,0051 |
| FBtr0088476 | lectin-46Ca | -4,4478 | -1,6056 | 0,0061 |
| FBtr0290073 | CG33287 | -4,3899 | -1,6151 | 0,0056 |
| FBtr0079797 | Ggamma30A | -4,3931 | -1,6216 | 0,0051 |
| FBtr0336963 | Ir92a | -4,3931 | -1,6216 | 0,0051 |
| FBtr0070764 | CanB | -4,3695 | -1,6417 | 0,0058 |
| FBtr0079305 | TTLL3A | -4,2953 | -1,6667 | 0,0052 |
| FBtr0299681 | CCKLR-17D1 | -4,2782 | -1,6667 | 0,0067 |
| FBtr0345656 | CG43187 | -4,2953 | -1,6667 | 0,0052 |
| FBtr0085754 | bnk | -4,2782 | -1,6667 | 0,0067 |
| FBtr0088113 | CG13223 | -4,2782 | -1,6667 | 0,0067 |
| FBtr0336953 | Ir67a | -4,3150 | -1,6780 | 0,0091 |
| FBtr0087071 | CG8910 | -4,3150 | -1,6780 | 0,0091 |
| FBtr0347504 | CG2528 | -4,2826 | -1,6818 | 0,0053 |
| FBtr0079432 | wg | -4,2826 | -1,6818 | 0,0053 |
| FBtr0070352 | Edem1 | -4,2367 | -1,7141 | 0,0076 |
| FBtr0085764 | ppk24 | -4,2367 | -1,7141 | 0,0076 |
| FBtr0074663 | CG14190 | -4,2367 | -1,7141 | 0,0076 |
| FBtr0073550 | Gr10b | -4,1836 | -1,7183 | 0,0061 |
| FBtr0303652 | CG15144 | -4,1478 | -1,7270 | 0,0095 |
| FBtr0081739 | CG10919 | -4,1848 | -1,7270 | 0,0053 |
| FBtr0086484 | hrg | -4,1498 | -1,7542 | 0,0068 |
| FBtr0074605 | CG12609 | -4,1498 | -1,7542 | 0,0068 |
| FBtr0345371 | CG13321 | -4,1498 | -1,7542 | 0,0068 |
| FBtr0273191 | CG10183 | -4,0731 | -1,7786 | 0,0059 |
| FBtr0335132 | CG3104 | -4,0533 | -1,7786 | 0,0077 |
| FBtr0074184 | CG32581 | -4,0533 | -1,7786 | 0,0077 |
| FBtr0346169 | CG32695 | -4,0533 | -1,7786 | 0,0077 |
| FBtr0300989 | CG32259 | -4,0731 | -1,7786 | 0,0059 |
| FBtr0088108 | CG13226 | -4,0533 | -1,7786 | 0,0077 |
| FBtr0309116 | CG14088 | -4,0520 | -1,7994 | 0,0063 |
| FBtr0075710 | CG17177 | -4,0520 | -1,7994 | 0,0063 |
| FBtr0074604 | Cyp308a1 | -4,0520 | -1,7994 | 0,0063 |
| FBtr0332191 | dpr19 | -4,0520 | -1,7994 | 0,0063 |
| FBtr0079604 | CG14274 | -4,0520 | -1,7994 | 0,0063 |
| FBtr0083541 | CG14317 | -4,0520 | -1,7994 | 0,0063 |
| FBtr0345543 | CG31955 | -3,9428 | -1,8388 | 0,0067 |
| FBtr0087780 | CG3955 | -3,9428 | -1,8388 | 0,0067 |
| FBtr0302430 | CG42263 | -3,9428 | -1,8388 | 0,0067 |
| FBtr0087577 | VGAT | -3,9428 | -1,8388 | 0,0067 |
| FBtr0076801 | CG14838 | -3,9428 | -1,8388 | 0,0067 |
| FBtr0081694 | Ccp84Ae | -3,9402 | -1,8510 | 0,0061 |
| FBtr0336964 | Ir11a | -3,9402 | -1,8510 | 0,0061 |
| FBtr0332502 | Pif1A | -3,9402 | -1,8510 | 0,0061 |
| FBtr0071734 | PpN58A | -3,9402 | -1,8510 | 0,0061 |
| FBtr0080990 | CG7094 | -3,9402 | -1,8510 | 0,0061 |
| FBtr0076587 | CG13312 | -3,9402 | -1,8510 | 0,0061 |
| FBtr0333336 | beat-VI | -3,9402 | -1,8510 | 0,0061 |
| FBtr0078605 | Osi13 | -3,9402 | -1,8510 | 0,0061 |
| FBtr0339524 | Qtzl | -3,9402 | -1,8510 | 0,0061 |
| FBtr0089045 | CG11060 | -3,9402 | -1,8510 | 0,0061 |
| FBtr0071969 | CG9899 | -3,9402 | -1,8510 | 0,0061 |
| FBtr0084345 | CG13837 | -3,9402 | -1,8510 | 0,0061 |
| FBtr0070555 | CG14422 | -3,9402 | -1,8510 | 0,0061 |
| FBtr0340114 | CG15602 | -3,9402 | -1,8510 | 0,0061 |
| FBtr0343296 | CG6106 | -3,9402 | -1,8510 | 0,0061 |
| FBtr0113232 | CCHa1 | -3,8848 | -1,8900 | 0,0092 |
| FBtr0087516 | phyl | -3,8848 | -1,8900 | 0,0092 |
| FBtr0088895 | Prosalpha1 | -3,8848 | -1,8900 | 0,0092 |
| FBtr0071150 | CG10920 | -3,8848 | -1,8900 | 0,0092 |
| FBtr0333136 | scrt | -3,8848 | -1,8900 | 0,0092 |
| FBtr0073497 | Sk1 | -3,8848 | -1,8900 | 0,0092 |
| FBtr0308027 | Ugt35E1 | -3,8100 | -1,9112 | 0,0064 |
| FBtr0084742 | CG13634 | -3,8100 | -1,9112 | 0,0064 |
| FBtr0113244 | CG14331 | -3,8100 | -1,9112 | 0,0064 |
| FBtr0082905 | CG31533 | -3,8100 | -1,9112 | 0,0064 |
| FBtr0083837 | CG4390 | -3,8100 | -1,9112 | 0,0064 |
| FBtr0304869 | FoxP | -3,7871 | -1,9112 | 0,0093 |
| FBtr0340231 | raw | -3,7871 | -1,9112 | 0,0093 |
| FBtr0343348 | CARPA | -3,8100 | -1,9112 | 0,0064 |
| FBtr0303149 | Gr47a | -3,8100 | -1,9112 | 0,0064 |
| FBtr0332385 | RunxB | -3,8100 | -1,9112 | 0,0064 |
| FBtr0110939 | SNF4Agamma | -3,8100 | -1,9112 | 0,0064 |
| FBtr0078291 | CG33284 | -3,8100 | -1,9112 | 0,0064 |
| FBtr0333135 | CG43915 | -3,8100 | -1,9112 | 0,0064 |
| FBtr0073774 | CG1640 | -3,8100 | -1,9112 | 0,0064 |
| FBtr0332105 | SPoCk | -3,7871 | -1,9112 | 0,0093 |
| FBtr0074796 | SkpD | -3,7871 | -1,9112 | 0,0093 |
| FBtr0081578 | Syt4 | -3,7871 | -1,9112 | 0,0093 |
| FBtr0112432 | CG34238 | -3,7871 | -1,9112 | 0,0093 |
| FBtr0332996 | CG13954 | -3,7728 | -1,9416 | 0,0078 |
| FBtr0072238 | CG4763 | -3,7728 | -1,9416 | 0,0078 |
| FBtr0081836 | Or85a | -3,7728 | -1,9416 | 0,0078 |
| FBtr0343684 | CG33310 | -3,7728 | -1,9416 | 0,0078 |
| FBtr0087925 | CG13157 | -3,7728 | -1,9416 | 0,0078 |
| FBtr0078503 | CG11449 | -3,7728 | -1,9416 | 0,0078 |
| FBtr0087080 | Acp53Ea | -3,6545 | -1,9836 | 0,0077 |
| FBtr0300486 | Ace | -3,6545 | -1,9836 | 0,0077 |
| FBtr0077423 | Bsg25A | -3,6545 | -1,9836 | 0,0077 |
| FBtr0091928 | CheB74a | -3,6545 | -1,9836 | 0,0077 |
| FBtr0332183 | OS9 | -3,6545 | -1,9836 | 0,0077 |
| FBtr0079281 | ppk7 | -3,6545 | -1,9836 | 0,0077 |
| FBtr0076069 | SdhAL | -3,6545 | -1,9836 | 0,0077 |
| FBtr0078233 | CG5282 | -3,6545 | -1,9836 | 0,0077 |
| FBtr0082951 | CG14841 | -3,6545 | -1,9836 | 0,0077 |
| FBtr0077286 | CG1314 | -3,6545 | -1,9836 | 0,0077 |
| FBtr0076329 | Ilp2 | -3,6545 | -1,9836 | 0,0077 |
| FBtr0290293 | Ir94f | -3,6545 | -1,9836 | 0,0077 |
| FBtr0077005 | Or65b | -3,6545 | -1,9836 | 0,0077 |
| FBtr0085929 | CG1428 | -3,6545 | -1,9836 | 0,0077 |
| FBtr0073051 | CG14960 | -3,6545 | -1,9836 | 0,0077 |
| FBtr0076576 | CG6576 | -3,6545 | -1,9836 | 0,0077 |
| FBtr0334692 | CG44038 | -3,6545 | -1,9836 | 0,0077 |
| FBtr0346123 | CG15743 | -3,6545 | -1,9836 | 0,0077 |
| FBtr0076981 | axed | -3,6425 | -2,0019 | 0,0076 |
| FBtr0346170 | CARPB | -3,6425 | -2,0019 | 0,0076 |
| FBtr0085934 | tplus3a | -3,6425 | -2,0019 | 0,0076 |
| FBtr0345622 | CG34193 | -3,6425 | -2,0019 | 0,0076 |
| FBtr0075511 | CG17032 | -3,6425 | -2,0019 | 0,0076 |
| FBtr0075101 | CG7271 | -3,6425 | -2,0019 | 0,0076 |
| FBtr0300330 | Pde6 | -3,4869 | -2,0742 | 0,0077 |
| FBtr0113295 | ppk15 | -3,4869 | -2,0742 | 0,0077 |
| FBtr0086341 | CG13872 | -3,4869 | -2,0742 | 0,0077 |
| FBtr0073373 | CG32232 | -3,4869 | -2,0742 | 0,0077 |
| FBtr0077006 | Or65c | -3,4869 | -2,0742 | 0,0077 |
| FBtr0080101 | CG17097 | -3,4869 | -2,0742 | 0,0077 |
| FBtr0091620 | CG33643 | -3,4869 | -2,0742 | 0,0077 |
| FBtr0081534 | CG14598 | -3,4869 | -2,0742 | 0,0077 |
| FBtr0083327 | CG14892 | -3,4869 | -2,0742 | 0,0077 |
| FBtr0082561 | CG3942 | -3,4869 | -2,0742 | 0,0077 |
| FBtr0310493 | CG43689 | -3,4869 | -2,0742 | 0,0077 |

**(b) The list of Su(Hw)^LOF^ down-regulated transcripts**

| ENSEMBLTRANS | SYMBOL | logFC | Average Expression | Adjusted  P-value |
| --- | --- | --- | --- | --- |
| FBtr0347286 | CG33946 | 5,0472 | 1,4039 | 0,0096 |
| FBtr0082044 | GstZ1 | 4,1492 | 4,0626 | 0,0042 |
| FBtr0073205 | dib | 4,0637 | 2,5742 | 0,0038 |
| FBtr0080500 | CG7110 | 3,9315 | -1,3456 | 0,0084 |
| FBtr0082707 | Hsc70-2 | 3,8866 | 5,6651 | 0,0076 |
| FBtr0085640 | CecB | 3,8743 | -1,3757 | 0,0077 |
| FBtr0091660 | CG33680 | 3,8119 | -1,4085 | 0,0071 |
| FBtr0345708 | CG18508 | 3,7888 | -1,4309 | 0,0078 |
| FBtr0346569 | CG10616 | 3,7734 | 0,7750 | 0,0072 |
| FBtr0075556 | yellow-k | 3,7278 | -1,4486 | 0,0084 |
| FBtr0304952 | TTLL4A | 3,5643 | -1,5299 | 0,0099 |
| FBtr0302355 | alpha-Est9 | 3,5588 | -1,5454 | 0,0087 |
| FBtr0340449 | tn | 3,4830 | -1,5815 | 0,0078 |
| FBtr0333344 | Hr4 | 3,3988 | -1,6216 | 0,0077 |
| FBtr0070664 | CG15571 | 3,3884 | 3,0376 | 0,0060 |
| FBtr0343637 | CG42598 | 3,3041 | -1,6667 | 0,0079 |
| FBtr0340028 | Top3beta | 3,2282 | 0,6145 | 0,0076 |
| FBtr0331990 | CG13398 | 3,1763 | 2,0662 | 0,0053 |
| FBtr0111144 | mRpS5 | 3,0542 | 2,7829 | 0,0052 |
| FBtr0084288 | CG33099 | 3,0387 | 4,1968 | 0,0051 |
| FBtr0309871 | HP1b | 3,0387 | 0,9542 | 0,0073 |
| FBtr0344354 | RhoGAP92B | 2,8332 | 0,6671 | 0,0095 |
| FBtr0083375 | EMC2A | 2,8131 | 2,9867 | 0,0065 |
| FBtr0331630 | cpb | 2,7745 | 2,6747 | 0,0051 |
| FBtr0087744 | GstE14 | 2,7560 | 4,7392 | 0,0055 |
| FBtr0342743 | wisp | 2,7003 | 4,0514 | 0,0073 |
| FBtr0087867 | nemy | 2,5317 | 3,1506 | 0,0072 |
| FBtr0083992 | CG17272 | 2,4946 | 6,3132 | 0,0035 |
| FBtr0346140 | CG1307 | 2,4385 | 2,8933 | 0,0052 |
| FBtr0300582 | CG42495 | 2,4351 | 3,1749 | 0,0053 |
| FBtr0079241 | CG13998 | 2,4262 | 3,4356 | 0,0059 |
| FBtr0089192 | Arf102F | 2,3805 | 3,2908 | 0,0051 |
| FBtr0337015 | CG14882 | 2,3686 | 1,8510 | 0,0092 |
| FBtr0086110 | sced | 2,3454 | 3,5494 | 0,0076 |
| FBtr0082853 | Rbp4 | 2,3381 | 2,0151 | 0,0076 |
| FBtr0078624 | jagn | 2,3140 | 3,9728 | 0,0061 |
| FBtr0346348 | RpII15 | 2,2251 | 2,5963 | 0,0072 |
| FBtr0077814 | CG15362 | 2,2052 | 4,2717 | 0,0081 |
| FBtr0085397 | stg | 2,1748 | 6,0414 | 0,0035 |
| FBtr0071249 | Hexo2 | 2,1581 | 6,2064 | 0,0076 |
| FBtr0112388 | CG34195 | 2,1495 | 5,7819 | 0,0038 |
| FBtr0334085 | CG7841 | 2,1386 | 2,8129 | 0,0064 |
| FBtr0340612 | Ubqn | 2,1305 | 7,3290 | 0,0067 |
| FBtr0083608 | CG7168 | 2,1173 | 5,4453 | 0,0073 |
| FBtr0079601 | strat | 2,0835 | 5,7276 | 0,0059 |
| FBtr0070987 | CG14440 | 2,0585 | 5,1263 | 0,0068 |
| FBtr0083713 | P5cr | 2,0350 | 6,2956 | 0,0061 |
| FBtr0080998 | GCS2beta | 2,0324 | 2,9733 | 0,0075 |
| FBtr0083913 | CG5466 | 2,0259 | 4,1314 | 0,0051 |
| FBtr0333492 | Ubc2 | 2,0181 | 4,6418 | 0,0051 |
| FBtr0087254 | Ric | 2,0069 | 3,8025 | 0,0094 |
| FBtr0334869 | Orct2 | 2,0015 | 3,1034 | 0,0092 |
| FBtr0079598 | CG7840 | 1,9992 | 7,8886 | 0,0038 |
| FBtr0079253 | Nepl4 | 1,9983 | 4,3047 | 0,0052 |
| FBtr0339820 | CG1677 | 1,9732 | 4,7591 | 0,0059 |
| FBtr0074686 | CG32536 | 1,9648 | 3,2679 | 0,0064 |
| FBtr0077154 | Pfdn4 | 1,9628 | 7,4710 | 0,0061 |
| FBtr0333748 | Tom40 | 1,9373 | 7,0777 | 0,0051 |
| FBtr0076396 | CG16711 | 1,9271 | 4,6524 | 0,0052 |
| FBtr0080232 | Ge-1 | 1,8888 | 3,4528 | 0,0063 |
| FBtr0333425 | CycY | 1,8831 | 4,5416 | 0,0096 |
| FBtr0081135 | EMC5 | 1,8609 | 6,4500 | 0,0038 |
| FBtr0071371 | CG15317 | 1,8448 | 5,6103 | 0,0077 |
| FBtr0344600 | RpII140 | 1,8356 | 3,8236 | 0,0059 |
| FBtr0333305 | flw | 1,8049 | 5,1341 | 0,0068 |
| FBtr0076511 | mRpL12 | 1,7959 | 8,8925 | 0,0051 |
| FBtr0087059 | ste24a | 1,7930 | 7,6645 | 0,0038 |
| FBtr0076984 | ndl | 1,7908 | 8,1359 | 0,0035 |
| FBtr0308699 | Top1 | 1,7892 | 6,0502 | 0,0079 |
| FBtr0087521 | CG8323 | 1,7788 | 4,4161 | 0,0051 |
| FBtr0346130 | CG43313 | 1,7757 | 4,6488 | 0,0049 |
| FBtr0075558 | CrebA | 1,7621 | 4,7011 | 0,0044 |
| FBtr0346163 | Rbm13 | 1,7559 | 4,7635 | 0,0069 |
| FBtr0080957 | CG12288 | 1,7483 | 8,0573 | 0,0038 |
| FBtr0088942 | mRpL52 | 1,7287 | 6,8461 | 0,0067 |
| FBtr0087586 | CG13344 | 1,7287 | 5,5847 | 0,0048 |
| FBtr0076689 | mus301 | 1,7261 | 5,5485 | 0,0042 |
| FBtr0081146 | CG10470 | 1,7107 | 7,6789 | 0,0038 |
| FBtr0088422 | Pfk | 1,7057 | 3,3079 | 0,0077 |
| FBtr0082969 | BigH1 | 1,7040 | 9,4713 | 0,0035 |
| FBtr0072904 | oxt | 1,7016 | 7,2773 | 0,0035 |
| FBtr0076393 | alphaTub67C | 1,6914 | 10,4708 | 0,0044 |
| FBtr0113361 | Ttd14 | 1,6880 | 6,6537 | 0,0059 |
| FBtr0084481 | CG10184 | 1,6853 | 7,0834 | 0,0035 |
| FBtr0307290 | east | 1,6652 | 4,9391 | 0,0059 |
| FBtr0084583 | Slimp | 1,6521 | 5,0069 | 0,0049 |
| FBtr0076613 | mtrm | 1,6349 | 8,9969 | 0,0044 |
| FBtr0076516 | CG5026 | 1,6318 | 3,2185 | 0,0077 |
| FBtr0308566 | wds | 1,6313 | 6,3229 | 0,0092 |
| FBtr0077768 | PIG-Wa | 1,6207 | 4,2284 | 0,0076 |
| FBtr0084285 | CG5326 | 1,6077 | 8,0841 | 0,0052 |
| FBtr0085458 | Pcd | 1,5938 | 7,3466 | 0,0038 |
| FBtr0081878 | SLIRP2 | 1,5823 | 3,7478 | 0,0088 |
| FBtr0113473 | nvd | 1,5791 | 4,2990 | 0,0077 |
| FBtr0088924 | CG12107 | 1,5722 | 8,3956 | 0,0035 |
| FBtr0082930 | PR-Set7 | 1,5655 | 5,3284 | 0,0099 |
| FBtr0086691 | PIG-O | 1,5641 | 6,7626 | 0,0038 |
| FBtr0082371 | ZnT86D | 1,5586 | 7,4590 | 0,0045 |
| FBtr0087261 | EMC7 | 1,5563 | 7,4998 | 0,0044 |
| FBtr0084797 | EMC6 | 1,5469 | 6,5310 | 0,0092 |
| FBtr0310001 | Afti | 1,5414 | 4,0346 | 0,0076 |
| FBtr0079937 | Pen | 1,5323 | 10,7028 | 0,0035 |
| FBtr0083283 | Manf | 1,5269 | 8,5654 | 0,0063 |
| FBtr0076879 | Srp19 | 1,5255 | 7,6367 | 0,0077 |
| FBtr0071039 | Setd3 | 1,5254 | 5,8523 | 0,0042 |
| FBtr0087498 | Sec61beta | 1,5195 | 9,5192 | 0,0042 |
| FBtr0084808 | Alg9 | 1,5189 | 6,8215 | 0,0070 |
| FBtr0113179 | Sgf11 | 1,5163 | 5,5557 | 0,0099 |
| FBtr0073926 | CG11674 | 1,5154 | 9,6381 | 0,0044 |
| FBtr0075453 | SsRbeta | 1,5134 | 9,4373 | 0,0046 |
| FBtr0075076 | CG3902 | 1,5043 | 9,3386 | 0,0061 |
| FBtr0083815 | CG11447 | 1,5041 | 5,4853 | 0,0045 |
| FBtr0073094 | CG17746 | 1,4951 | 3,8296 | 0,0092 |
| FBtr0072233 | Pym | 1,4940 | 6,6166 | 0,0038 |
| FBtr0273271 | Srp14 | 1,4928 | 7,3990 | 0,0077 |
| FBtr0345392 | CG8180 | 1,4918 | 5,3500 | 0,0045 |
| FBtr0073976 | CG12398 | 1,4881 | 7,5589 | 0,0090 |
| FBtr0084755 | beta4GalT7 | 1,4815 | 6,5685 | 0,0053 |
| FBtr0331967 | CG1344 | 1,4786 | 4,8613 | 0,0055 |
| FBtr0335518 | CG10979 | 1,4750 | 5,4631 | 0,0064 |
| FBtr0078198 | CG5969 | 1,4711 | 6,9318 | 0,0038 |
| FBtr0076241 | CG32069 | 1,4699 | 5,5095 | 0,0049 |
| FBtr0077460 | CG3652 | 1,4680 | 6,5385 | 0,0051 |
| FBtr0075067 | CG6843 | 1,4658 | 6,9623 | 0,0072 |
| FBtr0088058 | ERp60 | 1,4582 | 11,1809 | 0,0044 |
| FBtr0077025 | CG5568 | 1,4525 | 6,7369 | 0,0044 |
| FBtr0084870 | Sil1 | 1,4509 | 5,2738 | 0,0085 |
| FBtr0339838 | Rbp1-like | 1,4469 | 5,2170 | 0,0061 |
| FBtr0084671 | CG13625 | 1,4438 | 7,0155 | 0,0038 |
| FBtr0083993 | CG17271 | 1,4412 | 7,1646 | 0,0065 |
| FBtr0083539 | CG7785 | 1,4323 | 4,7565 | 0,0051 |
| FBtr0112557 | CG34348 | 1,4322 | 6,8699 | 0,0064 |
| FBtr0083316 | mRpS33 | 1,4268 | 6,9166 | 0,0077 |
| FBtr0075897 | mRpL20 | 1,4263 | 7,5868 | 0,0076 |
| FBtr0087840 | spt4 | 1,4240 | 6,3602 | 0,0044 |
| FBtr0087185 | fidipidine | 1,4240 | 6,0149 | 0,0051 |
| FBtr0113781 | Tim23 | 1,4239 | 9,3800 | 0,0035 |
| FBtr0100063 | CG34008 | 1,4231 | 4,3808 | 0,0060 |
| FBtr0083408 | Det | 1,4229 | 5,6606 | 0,0044 |
| FBtr0075766 | 26-29-p | 1,4222 | 11,2035 | 0,0092 |
| FBtr0084670 | Syx18 | 1,4115 | 6,3256 | 0,0045 |
| FBtr0089355 | lola | 1,4089 | 4,9243 | 0,0053 |
| FBtr0075457 | CG5027 | 1,4080 | 6,2986 | 0,0038 |
| FBtr0082172 | CG3909 | 1,4050 | 6,8048 | 0,0061 |
| FBtr0072207 | CG16787 | 1,3959 | 6,1974 | 0,0077 |
| FBtr0110879 | CG34132 | 1,3925 | 8,2813 | 0,0076 |
| FBtr0079540 | CCDC53 | 1,3829 | 4,3192 | 0,0074 |
| FBtr0077243 | Mgstl | 1,3804 | 5,5841 | 0,0052 |
| FBtr0079988 | CG4972 | 1,3766 | 8,5361 | 0,0035 |
| FBtr0082423 | CG14715 | 1,3714 | 8,4294 | 0,0068 |
| FBtr0300775 | Fdx2 | 1,3690 | 7,0960 | 0,0062 |
| FBtr0079682 | PIG-U | 1,3688 | 6,9414 | 0,0063 |
| FBtr0081099 | Grip71 | 1,3683 | 7,2721 | 0,0045 |
| FBtr0083909 | CG5412 | 1,3670 | 6,7110 | 0,0038 |
| FBtr0084576 | CG5510 | 1,3655 | 6,2536 | 0,0042 |
| FBtr0089007 | Eaf | 1,3653 | 4,6782 | 0,0091 |
| FBtr0086706 | CG5323 | 1,3637 | 7,7286 | 0,0076 |
| FBtr0332107 | Tudor-SN | 1,3587 | 5,5336 | 0,0064 |
| FBtr0075697 | gnu | 1,3549 | 9,1399 | 0,0044 |
| FBtr0088176 | dare | 1,3527 | 5,7951 | 0,0071 |
| FBtr0079960 | Snx17 | 1,3511 | 7,2205 | 0,0044 |
| FBtr0083682 | CG7718 | 1,3417 | 7,0724 | 0,0038 |
| FBtr0302226 | uex | 1,3380 | 3,5397 | 0,0091 |
| FBtr0086627 | MetRS | 1,3332 | 8,2207 | 0,0089 |
| FBtr0080122 | EMC3 | 1,3298 | 7,5667 | 0,0038 |
| FBtr0073338 | CG11342 | 1,3245 | 4,9046 | 0,0064 |
| FBtr0078348 | Aef1 | 1,3245 | 5,8885 | 0,0044 |
| FBtr0077391 | CG3036 | 1,3243 | 4,6093 | 0,0077 |
| FBtr0086306 | plu | 1,3224 | 5,8214 | 0,0051 |
| FBtr0077435 | Dim1 | 1,3194 | 6,3609 | 0,0094 |
| FBtr0075064 | Uggt | 1,3178 | 8,3141 | 0,0046 |
| FBtr0076000 | CG32099 | 1,3126 | 5,1564 | 0,0061 |
| FBtr0079027 | Rpn11 | 1,3119 | 9,2535 | 0,0052 |
| FBtr0080850 | CG5861 | 1,3095 | 7,6010 | 0,0038 |
| FBtr0084587 | eIF4EHP | 1,3095 | 5,8709 | 0,0099 |
| FBtr0084190 | Idh3b | 1,3064 | 6,7921 | 0,0063 |
| FBtr0084688 | Rpb10 | 1,3041 | 7,5901 | 0,0093 |
| FBtr0079403 | Sem1 | 1,3040 | 9,0614 | 0,0038 |
| FBtr0087852 | mRpL18 | 1,3029 | 7,2668 | 0,0052 |
| FBtr0073620 | FucT6 | 1,3000 | 6,6674 | 0,0056 |
| FBtr0078440 | CG7407 | 1,2988 | 5,6396 | 0,0048 |
| FBtr0085808 | CG11334 | 1,2959 | 3,7589 | 0,0081 |
| FBtr0070721 | CG6927 | 1,2905 | 7,6548 | 0,0099 |
| FBtr0075875 | CG11267 | 1,2885 | 10,3915 | 0,0085 |
| FBtr0087880 | CG8778 | 1,2868 | 7,8834 | 0,0051 |
| FBtr0343412 | l(1)G0320 | 1,2830 | 8,7112 | 0,0081 |
| FBtr0082406 | mgr | 1,2815 | 8,9456 | 0,0053 |
| FBtr0084968 | CG14543 | 1,2796 | 7,5603 | 0,0053 |
| FBtr0082715 | CG7966 | 1,2785 | 7,2928 | 0,0044 |
| FBtr0077964 | CG14341 | 1,2673 | 5,1282 | 0,0091 |
| FBtr0084213 | PyK | 1,2620 | 5,0308 | 0,0064 |
| FBtr0076750 | Srp9 | 1,2606 | 8,0216 | 0,0038 |
| FBtr0113217 | CG6567 | 1,2593 | 6,3809 | 0,0099 |
| FBtr0075643 | CG6878 | 1,2589 | 7,5544 | 0,0053 |
| FBtr0073505 | Uba5 | 1,2586 | 7,4687 | 0,0038 |
| FBtr0343116 | Jwa | 1,2577 | 8,3831 | 0,0083 |
| FBtr0071860 | bonsai | 1,2496 | 8,2934 | 0,0082 |
| FBtr0076653 | GstO2 | 1,2442 | 7,8024 | 0,0038 |
| FBtr0339155 | Patr-1 | 1,2441 | 4,4657 | 0,0077 |
| FBtr0078432 | Rpb8 | 1,2426 | 7,5764 | 0,0042 |
| FBtr0086899 | CG30105 | 1,2422 | 5,0952 | 0,0061 |
| FBtr0086832 | icln | 1,2402 | 7,9184 | 0,0038 |
| FBtr0079984 | CG4953 | 1,2398 | 5,6630 | 0,0069 |
| FBtr0083391 | CG5220 | 1,2353 | 7,3031 | 0,0072 |
| FBtr0086892 | P32 | 1,2337 | 9,8145 | 0,0044 |
| FBtr0071907 | EMC8-9 | 1,2317 | 7,1668 | 0,0071 |
| FBtr0089968 | Tm1 | 1,2310 | 6,1791 | 0,0057 |
| FBtr0075483 | PDCD-5 | 1,2306 | 7,7972 | 0,0075 |
| FBtr0339749 | CG1578 | 1,2271 | 5,4007 | 0,0064 |
| FBtr0074210 | Prosalpha4 | 1,2250 | 9,6205 | 0,0054 |
| FBtr0081687 | twr | 1,2217 | 7,2566 | 0,0076 |
| FBtr0088126 | san | 1,2206 | 8,2878 | 0,0042 |
| FBtr0084882 | msi | 1,2124 | 5,3424 | 0,0057 |
| FBtr0084758 | bai | 1,2106 | 8,7902 | 0,0054 |
| FBtr0086745 | CG10914 | 1,2052 | 6,3877 | 0,0089 |
| FBtr0081439 | Mtp | 1,1989 | 7,1364 | 0,0044 |
| FBtr0273404 | Tsp39D | 1,1963 | 6,0278 | 0,0052 |
| FBtr0074202 | CG11679 | 1,1939 | 6,5268 | 0,0077 |
| FBtr0078736 | CG31548 | 1,1936 | 7,9444 | 0,0063 |
| FBtr0080444 | mRF1 | 1,1927 | 6,0180 | 0,0054 |
| FBtr0083308 | Sdhaf3 | 1,1908 | 6,0907 | 0,0067 |
| FBtr0083781 | Nup58 | 1,1907 | 7,1303 | 0,0093 |
| FBtr0083616 | CG12321 | 1,1874 | 7,2551 | 0,0059 |
| FBtr0076737 | Arp3 | 1,1862 | 6,3273 | 0,0052 |
| FBtr0075645 | CG7011 | 1,1839 | 6,6081 | 0,0044 |
| FBtr0085776 | CG15561 | 1,1820 | 7,5210 | 0,0076 |
| FBtr0083373 | AdSL | 1,1796 | 8,6546 | 0,0035 |
| FBtr0071472 | Gip | 1,1680 | 7,8400 | 0,0069 |
| FBtr0081822 | CG2767 | 1,1680 | 6,9303 | 0,0063 |
| FBtr0073841 | Grip91 | 1,1658 | 6,7796 | 0,0044 |
| FBtr0084786 | Stt3B | 1,1657 | 9,1703 | 0,0064 |
| FBtr0076614 | Cbl | 1,1611 | 6,6441 | 0,0046 |
| FBtr0079613 | Ostgamma | 1,1588 | 8,2945 | 0,0035 |
| FBtr0078807 | CG11999 | 1,1586 | 6,8859 | 0,0044 |
| FBtr0087535 | Usp20-33 | 1,1569 | 7,1463 | 0,0059 |
| FBtr0078124 | mRpL10 | 1,1547 | 7,7327 | 0,0038 |
| FBtr0084452 | Rpt5 | 1,1434 | 9,2966 | 0,0067 |
| FBtr0337044 | mats | 1,1393 | 5,7203 | 0,0052 |
| FBtr0089318 | mRpL13 | 1,1376 | 7,0981 | 0,0082 |
| FBtr0078630 | CG1218 | 1,1372 | 7,3295 | 0,0061 |
| FBtr0078709 | CG12170 | 1,1331 | 6,4074 | 0,0052 |
| FBtr0085836 | CG1890 | 1,1326 | 8,3712 | 0,0079 |
| FBtr0078090 | CG13690 | 1,1284 | 6,8153 | 0,0076 |
| FBtr0084913 | CG14545 | 1,1260 | 9,1578 | 0,0053 |
| FBtr0081130 | msl-1 | 1,1245 | 5,1006 | 0,0062 |
| FBtr0083483 | CG31249 | 1,1213 | 6,2692 | 0,0061 |
| FBtr0077456 | MFS18 | 1,1213 | 6,4324 | 0,0052 |
| FBtr0086076 | CG30440 | 1,1206 | 5,9190 | 0,0064 |
| FBtr0078060 | shv | 1,1191 | 7,4406 | 0,0041 |
| FBtr0072208 | CG3803 | 1,1184 | 6,2426 | 0,0052 |
| FBtr0070993 | CG3226 | 1,1177 | 8,7892 | 0,0066 |
| FBtr0086674 | GstE6 | 1,1157 | 9,1664 | 0,0072 |
| FBtr0078080 | Spp | 1,1157 | 8,8599 | 0,0035 |
| FBtr0083667 | CG7706 | 1,1119 | 6,0673 | 0,0061 |
| FBtr0273238 | CG18190 | 1,1098 | 8,1886 | 0,0042 |
| FBtr0079975 | me31B | 1,1090 | 7,9891 | 0,0053 |
| FBtr0088463 | GstT1 | 1,1071 | 7,6574 | 0,0038 |
| FBtr0087262 | Prosbeta1 | 1,1049 | 9,1192 | 0,0077 |
| FBtr0089998 | Prosalpha7 | 1,1010 | 9,6721 | 0,0038 |
| FBtr0075629 | Prosbeta2 | 1,0980 | 9,5235 | 0,0044 |
| FBtr0079372 | CG13773 | 1,0965 | 7,7876 | 0,0071 |
| FBtr0088010 | SmF | 1,0954 | 8,7007 | 0,0052 |
| FBtr0076728 | Nmt | 1,0952 | 9,2085 | 0,0038 |
| FBtr0091612 | CG33635 | 1,0949 | 5,0798 | 0,0082 |
| FBtr0086696 | rswl | 1,0866 | 6,9716 | 0,0077 |
| FBtr0088478 | Marc | 1,0861 | 7,2671 | 0,0044 |
| FBtr0080228 | CG6230 | 1,0861 | 7,8413 | 0,0052 |
| FBtr0081981 | TrpRS | 1,0856 | 8,0816 | 0,0058 |
| FBtr0082849 | CG9588 | 1,0837 | 6,4975 | 0,0072 |
| FBtr0087738 | CG4627 | 1,0759 | 6,1009 | 0,0086 |
| FBtr0077192 | fliI | 1,0746 | 7,6077 | 0,0061 |
| FBtr0079049 | Gmd | 1,0698 | 7,0977 | 0,0051 |
| FBtr0072095 | Alg3 | 1,0643 | 6,5543 | 0,0052 |
| FBtr0087976 | 128up | 1,0637 | 9,2007 | 0,0064 |
| FBtr0080222 | CG6287 | 1,0633 | 10,4937 | 0,0044 |
| FBtr0340271 | CG30109 | 1,0600 | 6,7299 | 0,0087 |
| FBtr0073945 | mRpS25 | 1,0582 | 7,5376 | 0,0061 |
| FBtr0079029 | CG14036 | 1,0537 | 8,0740 | 0,0051 |
| FBtr0081177 | l(2)37Bb | 1,0488 | 7,3518 | 0,0045 |
| FBtr0085955 | Tif-IA | 1,0480 | 9,3179 | 0,0042 |
| FBtr0077080 | QC | 1,0468 | 6,5468 | 0,0071 |
| FBtr0075360 | Prosbeta6 | 1,0424 | 9,6437 | 0,0049 |
| FBtr0087206 | Rrp42 | 1,0419 | 7,2929 | 0,0044 |
| FBtr0079052 | Scox | 1,0400 | 7,9743 | 0,0068 |
| FBtr0083710 | CG14286 | 1,0375 | 6,8953 | 0,0045 |
| FBtr0079874 | Bka | 1,0368 | 7,3502 | 0,0039 |
| FBtr0089553 | Ubc10 | 1,0366 | 7,5507 | 0,0038 |
| FBtr0072706 | cue | 1,0356 | 8,9966 | 0,0044 |
| FBtr0082634 | trus | 1,0353 | 6,6628 | 0,0052 |
| FBtr0086945 | cyp33 | 1,0350 | 6,9006 | 0,0064 |
| FBtr0084493 | CG13599 | 1,0338 | 5,4991 | 0,0076 |
| FBtr0082974 | Rad17 | 1,0329 | 5,8209 | 0,0082 |
| FBtr0077693 | Elba2 | 1,0325 | 6,8900 | 0,0044 |
| FBtr0075952 | SmD1 | 1,0283 | 7,9544 | 0,0076 |
| FBtr0073159 | Sc2 | 1,0245 | 8,3406 | 0,0059 |
| FBtr0086502 | TBCB | 1,0232 | 7,0490 | 0,0052 |
| FBtr0083835 | mdlc | 1,0142 | 6,8069 | 0,0045 |
| FBtr0077189 | Rpt6 | 1,0117 | 9,2113 | 0,0049 |
| FBtr0075563 | CG16979 | 1,0109 | 6,6565 | 0,0077 |
| FBtr0088429 | Cdc2rk | 1,0101 | 6,1780 | 0,0059 |
| FBtr0070830 | GAA1 | 1,0097 | 7,8823 | 0,0038 |
| FBtr0079560 | SmE | 1,0078 | 8,9500 | 0,0091 |
| FBtr0083117 | Surf4 | 1,0055 | 8,5050 | 0,0046 |
| FBtr0084704 | CG7006 | 1,0048 | 8,0627 | 0,0042 |
| FBtr0080313 | Hacd1 | 1,0047 | 7,2122 | 0,0044 |
| FBtr0081897 | mRpS18A | 1,0032 | 7,7837 | 0,0065 |
| FBtr0075605 | CG7857 | 1,0027 | 6,4906 | 0,0064 |
| FBtr0300214 | ksh | 1,0022 | 7,3787 | 0,0042 |

**Supplementary Table 2. The statistics for the read alignments, mapped reads and valid Hi-C pairs generated in hicBuildMatrix 3.4.2 for raw contact matrices in the wild-type (WT) and Su(Hw)^LOF^ ovaries of egg chamber stages 1-8.**

|  | **WT** |  | **Su(Hw)^LOF^** | |
| --- | --- | --- | --- | --- |
|  | **replicate 1** | **replicate 2** | **replicate 1** | **replicate 2** |
| Sequenced reads | 137547510 | 128920446 | 111549056 | 165043480 |
| Min rest. site distance | 300 | 300 | 300 | 300 |
| Max library insert size | 1000 | 1000 | 1000 | 1000 |
| Pairs mappable and unique | 76434015 | 85425187 | 70143558 | 107439572 |
| Valid Hi-C contacts | 41830915 | 24469263 | 35895064 | 33871428 |
| One mate unmapped | 10968817 | 1182111 | 5062095 | 1937621 |
| One mate not unique | 2659770 | 2133378 | 2099812 | 3045495 |
| Low mapping quality | 47484908 | 40179770 | 34243591 | 52620792 |
| dangling end |  |  |  |  |
| self ligation (removed) |  |  |  |  |
| One mate not close to rest site |  |  |  |  |
| same fragment | 26162749 | 35836050 | 24542562 | 40685072 |
| self circle |  |  |  |  |
| duplicated pairs | 8440351 | 25119874 | 9705932 | 32883072 |

**Supplementary Table 3. Primers used for ChIP-qPCR experiments.**

| **primer** | **sequence** |
| --- | --- |
| 1A2 for | ACCACACATCAGTCATCGTGT |
| 1A2 rev | AGCATTCTTTTACCATGCGTAT |
| 50A for | TTGATAAATAGTCCAGCACGCATAC |
| 50A rev | ATACAAAGTGGTTTCAGCCAAGAAG |
| 62D for | TGATACCAGGCGAACAGAAATC |
| 62D rev | TTTGGGCTTGGTGAGAACAG |
| 66E for | AACTCCATTCCATTCACCTGTCTC |
| 66E rev | GCTGCTGATCCTCGCTTTCC |
| 87E for | TTTGCGTTTCGGCTGCTGTC |
| 87E rev | GGATGTTACATTGAGAGTGCTTAGG |
| 1A1 for | CTATTAAATGATTATCGCCCGATTACC |
| 1A1 rev | GCTTAGTGGATATACTCGTACATATAC |
| CG1677 rev | CTGCTGCACGCACACGAACA |
| CG1677 for | GCGTAAAGTGCCTGCCTGTC |
| CR43651 for | GTTTTATCATTTCACACACACACAACC |
| CR43651 rev | TTGCGCGCACACATACAAAA |
| Crol for | CCTCCTCGCTTTATTTTGTA |
| Crol rev | ACGCTACGTACAATTCCCAG |
| SMR rev | TCGTGCGCAGGAACGTTCGT |
| SMR for | CGGCACTCAGTTGCCAACGACTCTG |
